# A Multi-Population Consensus Genetic Map Reveals Inconsistent Marker Order among Maps Likely Attributed to Structural Variations in the Apple Genome

**DOI:** 10.1371/journal.pone.0047864

**Published:** 2012-11-05

**Authors:** Muhammad Awais Khan, Yuepeng Han, Youfu Frank Zhao, Michela Troggio, Schuyler S. Korban

**Affiliations:** 1 Department of Natural Resources & Environmental Sciences, University of Illinois, Urbana, Illinois, United States of America; 2 Key Laboratory of Plant Germplasm Enhancement and Specialty Agriculture, Wuhan Botanical Garden, Chinese Academy of Sciences, Moshan, Wuhan, People's Republic of China; 3 Department of Crop Sciences, University of Illinois, Urbana, Illinois, United States of America; 4 Istituto Agrario San Michele all'Adige Research and Innovation Centre, Foundation Edmund Mach, Trento, Italy; Washington State University, United States of America

## Abstract

Genetic maps serve as frameworks for determining the genetic architecture of quantitative traits, assessing structure of a genome, as well as aid in pursuing association mapping and comparative genetic studies. In this study, a dense genetic map was constructed using a high-throughput 1,536 EST-derived SNP GoldenGate genotyping platform and a global consensus map established by combining the new genetic map with four existing reliable genetic maps of apple. The consensus map identified markers with both major and minor conflicts in positioning across all five maps. These major inconsistencies among marker positions were attributed either to structural variations within the apple genome, or among mapping populations, or genotyping technical errors. These also highlighted problems in assembly and anchorage of the reference draft apple genome sequence in regions with known segmental duplications. Markers common across all five apple genetic maps resulted in successful positioning of 2875 markers, consisting of 2033 SNPs and 843 SSRs as well as other specific markers, on the global consensus map. These markers were distributed across all 17 linkage groups, with an average of 169±33 marker per linkage group and with an average distance of 0.70±0.14 cM between markers. The total length of the consensus map was 1991.38 cM with an average length of 117.14±24.43 cM per linkage group. A total of 569 SNPs were mapped onto the genetic map, consisting of 140 recombinant individuals, from our recently developed apple Oligonucleotide pool assays (OPA). The new functional SNPs, along with the dense consensus genetic map, will be useful for high resolution QTL mapping of important traits in apple and for pursuing comparative genetic studies in Rosaceae.

## Introduction

Genetic maps are routinely constructed and exploited for identifying marker-trait associations through quantitative trait loci (QTL) mapping. These maps play a critical role in contributing to our understanding of the genetic architecture of quantitative traits by providing information on number, strength, and mode of interaction of QTLs. Such knowledge provides insights into designing strategies for potential improvement of traits of interest via marker-assisted breeding (MAB) or map-based cloning of genes [1]–[3]. Availability of an accurate and high-resolution genetic map, densely populated with high-throughput co-dominant and reproducible molecular markers, enhances efficiency and likelihood of success of a QTL mapping effort. Earlier, it has been suggested that QTLs with moderate effects can be identified even with maps having fairly wide marker intervals (∼10 cM) [4], [5]. However, to avoid linkage drag while performing marker-assisted introgression or to side-step pursuing an additional step of fine-mapping to identify genes underlying a QTL, a well-saturated map is highly recommended [6]. Additionally, to run a quick QTL scan, a dense genetic map offers a choice of polymorphic markers for developing a genetic map in a new population with well-distributed markers. A saturated and accurate map with co-dominant, reproducible, and high-throughput markers not only properly localizes a QTL, but it can also yield an accurate estimate of the power of the QTL [6] and contributes to enhanced map resolution, transferability across laboratories and mapping populations, and to efficient genotyping.

Multiple genetic and physical maps have become available for many species, but these are of limited use for pursuing comparative studies as they are often developed based on a single specific population with novel molecular markers and segregation of novel phenotypes [7]. Often, these individual maps have a common set of co-dominant markers, used as anchor points, that aid in the process of integration to establish a consensus map for the target species [8], [9], [10]. Such bridging or intercross markers should be evenly distributed along a chromosome for proper integration. As different marker types of individual genetic maps are present at different frequencies within a genome, a consensus map will have finer resolution due to presence of combinations of marker types in such a single map. A consensus map enables localization and comparison of markers and QTLs that do not segregate in a single population with those of another population. This enables identification of homologous linkage groups, and allows for direct comparisons of QTLs identified in various genetic backgrounds [8], [10], [11]. Integration of multiple genetic maps results in enhanced genome coverage and alignment of order of markers along a linkage group, thus enabling identification of ambiguities and inconsistencies among maps, possibly due to either genotyping errors or structural variations in a genome.

Dense genetic maps have been constructed for several crops, and maps from multiple populations have also been integrated to establish consensus maps for some of these crops using conventional algorithms [12]. JoinMap [11] and Carthagène [13] are frequently used to combine datasets from multiple populations. Both softwares take into account sizes and structures of populations to estimate marker order and genetic distance using either common or bridge markers [7], [12], [14]. According to Yap et al. [7], these approaches are rather subjective, time-consuming, and often overlook hidden or lost inconsistencies and conflicts between maps. Also, missing values can negatively impact map integration. Based on a graphic scheme initially proposed by Yap et al. [7], a map integration method has proven useful in exposing and solving marker order problems across maps established from different populations of a species wherein genotypic data are not available. For this method, individual maps targeted for integration are first represented by directed acyclic graphs (DAG), and then these DAGs are merged together, based on shared vertices, to establish a consensus graph.. The directed cycle points out inconsistencies among maps, while nodes and edges represent mapped markers as well as defined order of adjacent markers, respectively [12]. Wu et al. [14] have developed a tool, designated as MergeMap, that utilizes a parsimonious approach to identify local reshuffles (inaccuracies in orders of nearby markers) and global displacements (markers with locations distant from correct positions), by removing the smallest set of marker occurrences, to resolve such conflicts. When genetic markers are shared by multiple individual maps, marker occurrence is defined as the appearance of a marker in an individual map. Therefore, deletion of a marker occurrence does not affect occurrences of the same marker in other maps [15]. Moreover, MergeMap depends on marker distances (in cM) in individual maps instead of genotype scores, and it resolves conflicts by identifying and removing marker occurrences from some maps after weighting marker order differences. For integration purposes, it is recommended to use reasonably reliable individual maps for the target species. According to Wang et al. [12], integration of multiple population maps seems straightforward, but in practice, chromosomal segmental duplication can result in multiple paralogous loci that complicate integration of maps. MergeMap has been successfully used in common bean (*Phaseolus vulgaris*), cowpea (*Vigna ungliculata*), barley (*Hordeum vulgare*), and rapeseed (*Brassica napus*) to establish consensus maps based on three, six, four, and three populations, respectively [12], [16]–[18].

Although several high-density apple genetic maps populated with different marker types (primarily SSRs, some SNPs, and a few SCARs) are available, these are based on different populations [6], [19], rendering them difficult to use for comparative studies. These include a genetic map for ‘Fiesta’ and ‘Discovery’ [20], [21], a genetic map for ‘Malling 9’ and ‘Robusta 5’ [22], an integrated physical and genetic map for ‘Co-op 16’ and ‘Co-op 17’ [19], and an integrated map based on six populations of apple [23]. Although these maps have common markers, these have been genotyped using different methods and different size populations. Moreover, there are some ambiguities regarding marker positions among these maps as reported by Han et al. [19] and Velasco et al. [23].

In this study, an apple genetic map has been constructed using a high-throughput SNP genotyping Illumina platform, and used to develop a consensus map for apple by combining all above reported maps. This has allowed for identifying conflicts in orders of loci among the different genetic maps, attributed to genomic structural variations, as well as to genotyping errors.

## Results

### Segregation features of a GoldenGate™ apple genotyping assay

**Figure 1:**
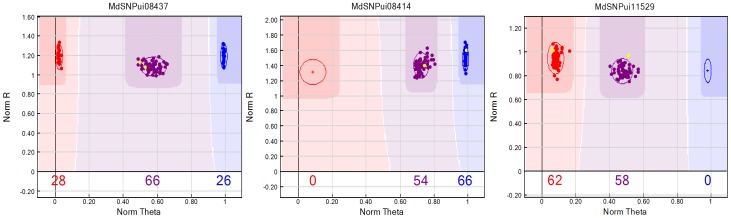
Genotyping plots of three SNPs showing segregation in Co-op 16 and Co-op 17 mapping population. Plots were generated by BeadStudio package (Illumina, San Diego, CA, USA) using normalized intensities of cy3 and cy5 flourescent dyes. The genotypes with intensities shown in red represents homozygous “aa”, purple represents heterozygous “ab”, blue stands for homozygous “bb” and yellow represents the genotypes for both parents. A) For “MdSNPui08437”, both parents are heterozygous “ab” and progeny plants are either homozygous “aa” or homozygous “bb” or heterozygous “ab” B) For “MdSNPui08414”, one parent is heterozygous “ab” while other is homozygous “bb” and progeny plants are either heterozygous “ab” or homozygous “bb” C) For “MdSNPui11529”, one parent is heterozygous “ab” while other is homozygous “aa” and progeny plants are either heterozygous “ab” or homozygous “aa”.

The oligonucleotide pool assays (OPAs) for apple consisted of 1536 SNPs containing 1411 genic SNPs, developed by Khan et al. [24], and 125 genomic SNPs, developed by Velasco et al. [23]. Of 1536 genotyped SNPs, 583 showed the expected segregation (1∶2∶1 or 1∶1) in the F1 apple mapping population (Figure 1, Table S1). There were 12 genomic and 116 genic SNPs with ab×ab segregation, 25 genomic and 203 genic SNPs with ab×aa (Co-op 17) segregation, and 33 genomic and 194 genic SNPs with aa×ab (Co-op 16) segregation. The genomic to genic SNP ratios were 1∶6 for Co-op 16 and 1∶8 for Co-op 17. In total, 56% of genomic SNPs segregated in the mapping population compared to 36% of genic SNPs. For each parent, 15% of SNPs and an additional ∼8% of SNPs, common to both parents, segregated in this mapping population. Overall, a total of 38% of SNPs from the GoldenGate™ apple genotyping assay segregated in this mapping population.

### New genetic map for Co-op 16 and Co-op 17

**Figure 2:**
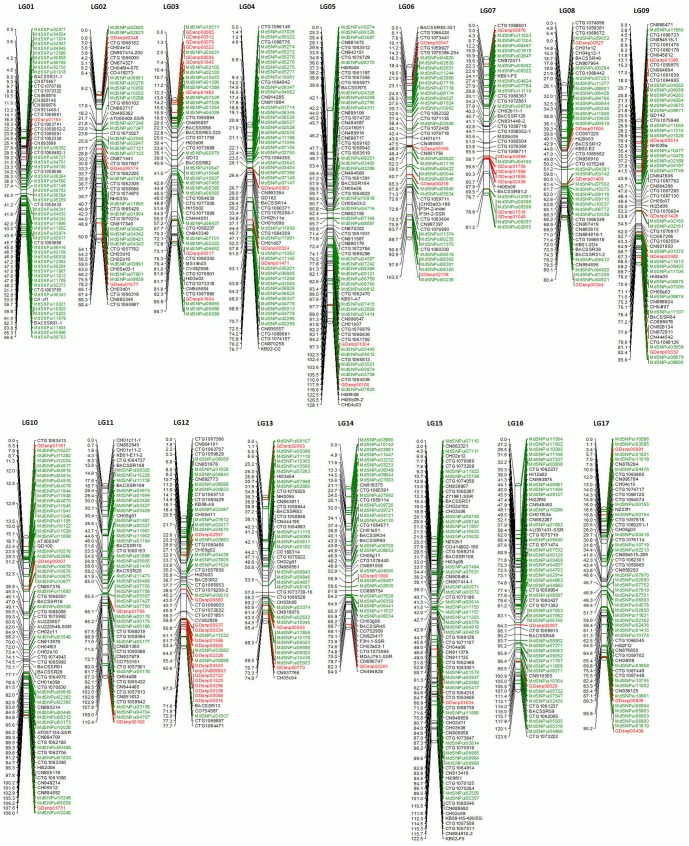
Genetic linkage map of apple showing 17 linkage groups, developed for F1 cross between ‘Co-op 16’ and ‘Co-op 17’. Markers in green font are genic SNPs from Khan et al. [6], markers in red font are genomic SNPs from Han et al. [23] and markers in black font are those genotyped by [19].

Following linkage analysis using 583 SNPs segregating in the mapping population along with 447 SSR markers previously used by Han et al. [19] for constructing an integrated physical and genetic map, 17 dense linkage groups were obtained (Figure 2). As 14 markers showed problems in linkage analysis, these were removed, yielding a final genetic map of 1016 markers, consisting of 569 new SNPs along with 447 markers from Han et al. [19]. Of the newly mapped SNPs, 499 were genic (EST-derived) and 70 were genomic [23]. Most SNPs mapped to their corresponding linkage group, as predicted by similarities of SNP sequences to genomic sequences.

On average, there are 60±11 markers per linkage group and the average interval between markers is ∼1.54±0.28. The total linkage group length is 1537.73 cM, with an average of 90.54±15.20 per linkage group. The longest linkage group is LG 15 (122.48 cM), while the shortest is LG 01 (66.62 cM) (Table 1).

**Table 1:** Features of the new genetic map of apple for Co-op 16×Co-op 17 constructed using SNP OPA designed by [6] and together with markers from [19].

Linkage group (LG)	Number of markers	Average interval per LG (cM) ± Standard deviation	Maximum interval (cM)	Linkage group length (cM)
LG01	66	1.01±1.20	6.19	66.62
LG02	56	1.58±1.91	8.38	88.42
LG03	58	1.50±1.82	10.46	86.72
LG04	65	1.18±1.54	7.25	76.69
LG05	75	1.35±1.49	6.91	101.45
LG06	51	2.03±2.09	7.76	103.52
LG07	41	1.98±2.12	9.25	81.24
LG08	53	1.52±1.71	8.54	80.43
LG09	68	1.26±1.26	7.51	85.55
LG10	75	1.44±1.64	7.38	107.96
LG11	57	2.02±2.27	9.24	115.37
LG12	58	1.34±1.75	10.38	77.68
LG13	48	1.56±2.78	18.62	74.83
LG14	47	1.79±1.77	6.87	83.96
LG15	80	1.53±1.65	9.44	122.48
LG16	59	1.60±1.44	5.58	94.55
LG17	59	1.53±1.91	7.33	90.24
Total	1016			1537.73
Average	60±11	1.54±0.28		90.45±15.20

The number of markers, average interval (cM), maximum interval (cM) per linkage group and length (cM) of each linkage group are shown.

### Global consensus genetic map of apple

The parental maps from earlier studies [21] and our newly constructed map described above were successfully merged to construct a consensus map for apple (Table 2, Figure S2). This was achieved due to presence of multiple common markers across all five maps (Table 2). In total, there were 289 markers in common across at least two maps with 766 anchor points. There were 147 anchor points between ‘Fiesta’ and ‘Discovery’ maps, along with 144, 128, and 107 anchor points among ‘Discovery’ and ‘Fiesta’ together, ‘Co-op 16’×‘Co-op 17’, and the integrated map by Velasco et al. [23], respectively. There were only 18 anchor points between ‘Co-op 16’×‘Co-op 17’ and ‘M9’×‘R5’ maps. The highest number (79) of anchor points was detected on linkage group 10, while the lowest (22) was detected on LG 08. The ‘Fiesta’, ‘Discovery’, and ‘M9’×‘R5’ maps did not have any anchor points for some of the linkage groups, and in most cases with the ‘Co-op 16’×‘Co-op 17’ map. The consensus map consisted of 2875 markers, primarily consisting of SSRs and SNPs, with a few SCAR markers. The majority of these markers originated from apple, along with a few markers from pear.

**Table 2:** The common markers across different linkage groups and genetic maps used to construct a consensus genetic map of apples showing the anchor points between pair of genetic maps and corresponding linkage groups, as well as the total number of markers in common on each linkage group.

Maps	LG01	LG02	LG03	LG04	LG05	LG06	LG07	LG08	LG09	LG10	LG11	LG12	LG13	LG14	LG15	LG16	LG17	Anchors/Map
Co-op 16×Co-op 17_Discovery	1	3	3		2				3	4		1	2	2	5	1	1	28
Co-op 16×Co-op 17_Fiesta		4	3	1	2		1	1	2	5		2	3	2	4	3	2	35
Co-op 16×Co-op 17_M9×R5	1	3		1	1		1		2	1		1	4		1	1	1	18
Co-op 16×Co-op 17_Integrated	2	8	11	4	5	4	9	5	8	7	2	17	8	4	6	3	4	107
Discovery_Fiesta	6	8	6	7	12	7	4	4	7	18	10	11	7	12	11	9	8	147
Discovery_M9×R5	2	5	2	2	3	2	2	1	2	4	3	6	4	1	5	5	2	51
Discovery_Integrated	6	8	6	7	15	4	4	5	7	17	9	12	9	14	10	8	3	144
Fiesta_M9×R5	2	5	2	3	3	2	3	2	2	4	3	6	4	1	4	5	3	54
Fiesta_Integrated	6	10	4	9	10	4	4	3	6	16	8	11	6	11	8	8	4	128
M9×R5_Integrated	3	5	2	3	3	2	2	1	3	3	3	6	6		4	5	3	54
Anchors/LG	29	59	39	37	56	25	30	22	42	79	38	73	53	47	58	48	31	766
Markers/LG	11	18	18	16	21	11	14	13	14	26	15	31	18	20	15	15	13	

Fiesta and Discovery are ‘Fiesta’ and ‘Discovery’ maps [21], M9×R5 map [22], Integrated is the integrated map based on six populations [23] and our newly constructed map (Co-op 16×Co-op 17) wherein LG stands for linkage group.

The consensus map consists of both EST-based and genomic SSRs and SNPs. On average, there are 169±33 markers/linkage group. The linkage group with the lowest level of polymorphism is LG 11, having 180 markers distributed along 172.75 cM; while LG 01 has the highest level of polymorphism with 167 markers within a length of 85.33 cM. The average interval between markers is 0.70±0.14 cM, and the longest interval, of 27.16 cM, is on LG 16. The total length of linkage groups is 1991.38 cM with an average of 117.14±24.43 cM. LG 01 is the shortest (85.33 cM), while LG 11 is the longest (172.75 cM). When estimated by Fishman et al. [25] and method 4 of Chakravarti et al. [26], lengths of linkage groups are highly similar to corresponding linkage groups of the consensus map. Genome coverage estimation shows that the constructed consensus map covers ∼99% of the apple genome (Table 3). The linkage group length of the consensus genetic map is inflated, and the scaling factor is estimated at 0.63±0.12.

**Table 3:** The number of markers, average interval (cM) ± standard deviation, maximum interval (cM) per linkage group, length (cM) of each linkage group of the consensus map of apple, and genome coverage (%) per linkage group.

Linkage group (LG)	Number of markers	Average interval per LG (cM) ±Standard deviation	Maximum interval (cM)	Linkage group length (cM)	Average Ge per LG*	Genome Coverage (%) per LG*
LG01	167	0.51±0.59	2.85	85.33	86.35	0.99
LG02	210	0.64±1.41	14.68	135.40	136.69	0.99
LG03	172	0.64±1.00	8.30	110.25	111.54	0.99
LG04	160	0.64±0.86	4.76	102.30	103.58	0.99
LG05	190	0.75±1.93	24.37	142.30	143.79	0.99
LG06	131	0.81±1.06	6.08	105.62	107.24	0.98
LG07	108	0.76±1.11	7.15	81.68	83.20	0.98
LG08	161	0.59±0.71	4.78	95.63	96.82	0.99
LG09	188	0.52±0.64	3.76	97.51	98.55	0.99
LG10	170	0.71±0.79	5.25	120.03	121.45	0.99
LG11	180	0.96±1.85	18.02	172.75	174.67	0.99
LG12	174	0.64±0.88	5.39	111.53	112.82	0.99
LG13	152	0.91±2.31	26.29	137.84	139.66	0.99
LG14	140	0.72±1.05	6.65	101.22	102.67	0.99
LG15	261	0.55±0.77	5.70	143.67	144.77	0.99
LG16	152	0.91±2.32	27.16	139.13	140.95	0.99
LG17	159	0.69±1.32	11.00	109.19	110.57	0.99
**Total**	2875			1991.38		
**Average**	169±33	0.70±0.14		117.14±24.43		

The consensus map was constructed by merging ‘Fiesta’and ‘Discovery’ maps [21], the genetic map for M9×R5 [22], an integrated map based on six populations [23], and our newly constructed map of Co-op 16×Co-op 17. The average Ge per LG is the average estimated genome length per linkage group calculated using the method of Fishman et al. [25] and method 4 of Chakravarti et al. [26]. Genome coverage (%) per LG was calculated by dividing the observed linkage group length by the estimated genome length of the corresponding LG multiplied by 100.

### Conflicts in order of markers among genetic maps of apple

A total of 58 markers showed conflicts among different maps and were removed by MergeMap (Table 4). Among these, there were 14 markers whose forward primer sequences along with eight markers whose reverse primer sequences did not show any significant similarities to the apple genome sequence. Five markers, including three markers originating from pear (NH029a, NH009b, and KA4b) did not show any significant similarities for either forward or reverse primers. Furthermore, among these 58 markers, forward primer sequences of 10 markers showed similarities with more than one linkage group, while eight reverse primer sequences showed similarities with more than one linkage group. Additionally, seven SNP markers from the map of Velasco et al. [23] showed similarities with more than one linkage group. A total of nine markers were removed from LG 13, seven markers were removed from LG 02, and five markers from each of LGs 05 and 12 were removed. No marker was removed from LG 06. There were only six markers that were removed that were present in only a single map, while all others were common to more than one map.

**Table 4:** Markers with conflicting positions across different studies identified and removed by MergeMap [14] during the construction of a consensus map for apple.

Marker	Map	Sequence of SNP/forward primer sequence for SSR*	Reverse primer sequence for SSR*	Number of Maps**	Multilocus***
CH03g12	M9×R5	(01), (03)	(01), (03)	(01)1	CH03g12b, (3)1 CH03g12y, (3)3 CH03g12z, (1)3
KA4b	M9×R5, Discovery			(01)4	
CN581493-SSR	Discovery	(02)2	(02)2	(02)2	
Hi24f04	Discovery		(14)	(02)3	
CH02c06	Fiesta			(02)3	
CN493139-SSR	M9×R5	(02)3, (08), (15)	(02)2, (05)2	(02)1	CN493139-SSR, (2)2 CN493139_3, (2)1 CN493139_5, (2)1
Hi07d12	M9×R5	(02)4, (09)2, (11), (15)2, un	(02)3	(02)2	Hi07d12x, (7)1
Hi05g12	M9×R5	01, (03)3, (10)2, (12)2, 14, un	(01), (03), (10)3, (12)	(02)3	
CH03d10	Integrated	(02)	(02)	(02)5	
Hi03e03	Discovery	(03)	(03)	(03)3	
GDsnp00506	Co-op 16×Co-op 17	(03)		(03)2	
HGA8bx	Fiesta	(11)	(03)2, (11), (14)	(03)2	HGA8by, (11)1
GDsnp00322	Integrated	(03)		(03)2	
CH01d03	Fiesta		(04)	(04)2	CH01d03, (4)1 CH01d03z, (12)2
Hi08e04	M9×R5, Integrated		(04)7	(04)4	Hi08e04a, (4)1
CH02c02b	Integrated		(04)2	(04)3	CH02c02a, (2)2
CN496002-SSR	Fiesta	(05)	(05)	(05)2	
CH04e03	Fiesta	(05)	(05)	(05)4	
Hi21c08	Integrated	(05)2, (10)		(05)2	
CH03a04	Integrated	(05)	(05)	(05)3	
Hi02a03	Integrated		(09)	(05)3	
GDsnp02436	Co-op 16×Co-op 17	(01), (07)		(07)2	
GDsnp00699	Integrated	(07), (15)2		(07)2	
GDsnp01756	Integrated	(07)2, un		(07)2	
CH02g09	Fiesta	(08)	(08)	(08)3	
GDsnp01048	Integrated	(07), (08)3, (15)3		(08)1	
GDsnp01370	Integrated	(08)		(08)1	
GDsnp02037	Integrated	(08)2		(08)1	
NH029a	Co-op 16×Co-op 17			(09)3	
Hi04a05	Fiesta	(01)	(01)	(09)3	
ch05c07	M9×R5	(09)	(09)	(09)5	
MS02a01	Discovery	(10)4		(10)3	
CH02a08	Fiesta	(10)3, un	(10)3	(10)3	CH02a08, (10)1 CH02a08z, (5)3
Hi07g10	Fiesta	(09)3, (13)3, 15, (un)3	(05)2, (10), (11), (13)2, (17), (un)3	(11)2	
CH04a12	Fiesta	(03)2, (11)2	(11)2	(11)3	
Hi02c06	Integrated		(13)	(11)3	
CH01f02	M9×R5	(12)	(12)	(12)4	
GDsnp01798	Integrated	(04), (12)		(12)1	
GDsnp00338	Integrated	(04)2, (12), un		(12)2	
GDsnp02228	Integrated	(12)		(12)2	
CH05g07	Integrated, Fiesta	(14)4	(14)3	(12)3	CH05g07, (12)1 (14)1 CH05g07z, (14)3
GDsnp00770	Co-op 16×Co-op 17	(13)		(13)2	
CH05h05	Co-op 16×Co-op 17	(13)	(13)2	(13)4	
CH01b12	Discovery	(03)2, (04), (10), (12)2, (17)	(12)3, (16)	(13)3	CH01b12x, (4)2 CH01b12z, (12)2
CH03h03	Fiesta	(10), (12)2, (15), un	(10), (12), un	(13)2	CH03h03, (13)1
NH009b	Fiesta			(13)4	
CH03a08	Fiesta	(13)2	(13)2	(13)4	
Hi03e04	Fiesta		(13)	(13)5	
Hi04g05	M9×R5		(13)	(13)3	
CH05c04	M9×R5, Co-op 16×Co-op 17, Integrated		(13)3	(13)4	CH05c04_4, (13)1
CH01e01	Fiesta	(un)2		(14)3	
NZ02b01	Discovery	(15)2	(15)2	(15)4	
Z71981-SSR	Discovery	(15)2	(15)2	(15)4	
Hi03f09	Fiesta			(15)2	
CH04f10	Fiesta	(16)	(16)	(16)4	
CH02d10a	Integrated	(16)	(16)2	(16)3	CH02d10b, (15)1
GDsnp00809	Co-op 16×Co-op 17	(6), (17)2		(17)2	
CH02g04	M9×R5	(17)	(09), (12), (17)4, (un)2	(17)3	

Conflicts in marker position in these markers could be attributed to technical errors and the segmental duplication in apple genome. The name of marker, map, linkage group according to marker sequence similarity based on apple genome sequence, number of maps that carry this marker, and multi-locus status is given in the table. Similarity is reported if *e*-values of the marker sequence are more than 0.01.

Note:

* Sequence similarity of SNP and SSR forward and reverse primers against the apple draft genome sequence. Number in parenthesis represents the linkage group(s). Multiple regions on the same linkage group showing similarity (*e*-value >0.01) are shown by the number outside the parenthesis. The abbreviation ‘un’ stands for unanchored sequence.

** Number within parenthesis is linkage group while outside is how many maps have this marker. The abbreviation ‘un’ stands for un-anchored sequence.

*** Represent multiple loci amplified by one marker; number in parenthesis is the linkage group, while number outside of the parenthesis is the number of maps wherein this marker is present.

Of all 58 markers removed, the highest number of markers removed from any single map was 18 markers from the ‘Fiesta’ map [21]. In total, there were 179 markers on the ‘Fiesta’ map that were common to other maps, thus 10% of markers were removed due to inconsistencies. Among 18 markers removed from the ‘Fiesta’ map, four were from LG 13. Whereas, only eight markers (4%) were removed from a total of 188 markers from the ‘Discovery’ map [21], common to other maps. The highest number of markers removed due to discrepancies in order of markers among maps, 11 (18%) out of 60 markers common to all other maps, was from the ‘M9’×‘R5’ map. Of 244 markers common to all maps, a total of 19 markers (8%) were removed in the map of Velasco et al. [23]. Among these 19 markers, four markers were located on LG 12.

The following six markers, Hi07d12, CH01d03, CH02c02b, CH02a08, CH05g07, and CH02d10a, were multi-allelic, and mapped onto multiple linkage groups. Their primer sequences showed similarities with sequences on the apple genome sequence for some chromosomes corresponding to mapped linkage groups, but not to all corresponding linkage groups. Markers Hi24f04, Hi02a03, Hi04a05, and Hi02c06 showed sequence similarities to a chromosome different from their corresponding linkage groups. Among these four markers, Hi02a03 and Hi02c06 were mapped onto the linkage map of Velasco et al. [23]. The forward primer sequence of CH01e01 had significant sequence similarity with an unanchored contig, and it was mapped onto linkage group 14 in three genetic maps, including that of Velasco et al. [23]. Marker CH03h03 was mapped only onto LG 13 in three maps, yet neither forward and reverse primer sequences showed any significant sequence similarities to chromosome 13.

## Discussion

### The GoldenGate™ apple genotyping assay and the new genetic map

The recently developed apple OPA [6] proved to be very useful in constructing a new map for apple. Of 1536 SNPs, 583 SNPs segregated in the mapping population of ‘Coop 16’×‘Coop 17’ while the remaining SNPs were either derived from duplicated regions, as predicted by Khan et al. [6], or were non-polymorphic, and hence failed to segregate. The high number of SNPs fitting the expected segregation ratio, even though the pedigrees of both ‘Co-op 16’ and ‘Co-op 17’ have common ancestors [27], suggests that this OPA will be even more useful in a cross between genetically diverse parents. This high frequency of observed polymorphism is due to the fact that the OPA is predominantly based on SNPs derived from EST sequences of 14 diverse apple genotypes [6]. Generally, EST sequences tend to be more conserved compared to genomic sequences, thus EST-derived SNPs are more likely to be transferable and with lower polymorphisms. Thus, it can be expected that SNPs identified from non-genic genomic sequences of the same 14 genotypes are likely to exhibit higher polymorphisms. However, there is a likelihood of either failure or amplification problems in genomic SNPs due to the fact that genomic sequences are more diverse than genic sequences. In this study, both parents show similar numbers of segregating markers (∼15%) in their progeny, but there is a higher ratio of genic (1∶6) to genomic SNPs in ‘Co-op 16’ compared to ‘Co-op 17’. This observed difference would suggest that there is a higher level of polymorphism in genomic regions of ‘Co-op 16’. As the frequency of markers with common alleles from both parents and those segregating in the progeny is ∼8%, this provides a baseline for anchoring both parental genotypic datasets and for constructing an integrated map.

The newly constructed map has a total of 1016 genic and genomic SNPs and SSRs, with additional 569 SNPs, compared to our previously constructed integrated map [19], distributed over all 17 linkage groups of apple. As the new SNPs are derived from expressed sequences, they can provide direct functional interpretation of any marker-trait associations identified. Although genic SSRs are already present in published apple genetic maps [19], genic SNPs will not only increase the number of functional markers for apple, but will also be advantageous over SSRs due to availability of high-throughput SNP genotyping assays. Presence of 70 SNPs from Velasco et al. [23] in this newly constructed map also enhances comparisons of the apple genome sequence and genetic maps of apple [6], particularly for establishing corresponding linkage groups. Moreover, these markers could be used as anchors to investigate sequences underlying QTL markers in future linkage studies. Due to the high density of markers, with an average interval of ∼1.54±0.28 between markers, this newly constructed map is well-suited for high-resolution QTL mapping. The observed small interval between markers can be attributed to presence of both SNPs and SSRs in this map. As different marker types have different frequencies within a genome, combining them increases the total frequency of markers within a given genome. For instance in plants, there is one SSR per 6 kb [28]; whereas, the frequency of SNPs within a genome is much higher, in the order of 100 bp. When using this map, any identified marker-trait association will have on average a reproducible marker at less than 2 cM on either side of the target locus. This high density of markers, along with presence of SSRs from BACs, will significantly reduce the time and cost of laborious fine-mapping studies. There is a 18.62 cM gap in LG 13. This gap may be attributed to low levels of polymorphism in this linkage group.

### Features of the consensus genetic map of apple

Four high-quality maps, together with the newly constructed genetic map developed in this study, were successfully merged to construct a consensus map for the apple genome. Successful merging of these maps was made possible by the presence of multiple common markers across all five maps. In apple, the genetic map constructed by Liebhard et al. [20] and its updated version [21] has long served as a reference, and SSR markers along this map have been used in most subsequent linkage map construction studies. For this reason, many linkage maps of apple have markers in common, providing a basis for pursuing comparative QTL analysis.

In this study, maps selected for constructing a consensus map for apple met criteria for successful merger. The maps are predominantly based on SSR and SNP markers, that are robust and less prone to genotyping errors than other marker types, and have multiple markers in common, a prerequisite for merging maps. The genetic maps of ‘Fiesta’ and ‘Discovery’ [21] are enhanced from the reference map developed by Liebhard et al. [20]; while the genetic map of ‘M9’×‘R5’ [22] is of apple rootstocks. The integrated map based on six populations (developed by Velasco et al. [23]) has been used to anchor the apple genome sequence, and the ‘Co-op 16’×‘Co-op 17’ map of Han et al. [19], now including new SNPs, is an integrated physical and genetic map anchored by BACs.

In total, there are 289 markers common across at least two maps, with a total of 766 anchor points (Table 2). The highest number of common markers is between ‘Fiesta’ and ‘Discovery’ maps, followed by the ‘Co-op 16’×‘Co-op 17’ map, and then the map of Velasco et al. [23]. Hence, integration among these maps should be highly reliable, and they are well-suited for the development of a comprehensive consensus map for apple. However, some linkage groups on ‘Fiesta’, ‘Discovery’, and ‘M9’×‘R5’ maps do not have markers in common with many linkage groups of the ‘Co-op 16’×‘Co-op 17’ map. Therefore, integration among these linkage groups, particularly among maps with fewer common markers, is likely to be poor. There are 2877 markers on the consensus map for apple, the majority of which are genomic and genic SSRs and SNPs (Figure S2). SSRs are highly useful in conducting comparative genomics studies among diverse germplasm, and even across different species [2], [29]. Presence of both genic and genomic markers will also provide insights into evolutionary relationships, as well as evolution of important functionally relevant regions within a genome [6], [30].

This consensus map sheds some light on various features of apple chromosomes. For example, presence of 169±33 markers/linkage group with a marker interval of 0.70±0.14 cM provides an excellent framework for selecting well-distributed and robust markers to construct a genetic map in any mapping population of apple. LG 11 is the longest and has the lowest number of markers/cM (1 marker/cM), thus indicating that there is low polymorphism in this linkage group compared with others. Whereas LG 01, the shortest linkage group, has the highest number of markers/cM (2 markers/cM), indicating incidence of high levels of polymorphism in this linkage group. Based on different methods of genome coverage estimation [25], this consensus map covers ∼99% of the apple genome. Moreover, this high estimation of genome coverage provides confidence that markers selected from this map are well positioned to identify various target genes and/or QTLs within the apple genome, and will also facilitate comparisons of QTLs across different studies. Additionally, many of the markers positioned along the consensus map are also located on different pear genetic maps, and some of the SSRs originating from pear are also present in this consensus map. Therefore, due to the high synteny between apple and pear [29], this map will also be useful in comparing QTLs from mapping studies in apple and pear.

It is important to point out that the consensus map is more relevant for positioning of the order of markers than absolute distances between markers. On average, the length of linkage groups of the consensus genetic map is inflated by a scaling factor of 0.63±0.12. MergeMap assigns bins to markers by estimating distances between them using a marker in common across maps. According to Close et al. [18], when two or more maps from different mapping populations are compared, recombination frequencies are not proportional to physical distances nor are they consistent. Therefore, DAGs in MergeMap provide a more accurate description of limitations of marker order than a linear map derived using approximations based on recombination values. Moreover, the consensus map based on merging different maps is simply one of many possible non-conflicting linear representations of the consensus DAGs [18]. Therefore, marker order in the consensus map will not perfectly match the order of corresponding nucleotides in a genome sequence. As marker order and distances (in cM) of individual maps are used in MergeMap instead of genotypic data of individual populations, localized errors in the consensus map may be present due to reversal of locations for two adjacent markers. However, order of markers at longer distances should most often be correct. In this study, maps merged to construct the consensus map are of good quality and are based on robust reproducible markers; thus, marker order in the consensus map should be accurate. In the future, data from additional mapping populations will increase the numbers of shared markers among maps, resulting in finer resolution and a more correct ordering of all markers located on this consensus map [18], [31].

### Inconsistencies in marker order between maps

During construction of the consensus map, MergeMap identified 58 markers (Table 4, Figure S1) that showed conflicts in marker order between different maps, and hence these were removed. The BLAST search of forward and reverse sequences of 45 SSRs and sequences of 13 SNPs against the apple genome sequence enabled prediction of the causes for this conflicting order of markers. Our results have suggested multiple reasons for this observed finding. These inconsistencies in marker order could be due to either technical errors from genotyping methods or biological factors such as local and segmental duplications or polyploidy events [19], [23]. These biological factors might have caused conflicts in marker order as follows. They might have resulted in repetitive sequences in multiple locations in the genome, thus increasing errors in genotyping, similar to those encountered by multi-locus markers. Moreover, local and segmental duplications or polyploidy events could have served as hot spots of structural variations, thus influencing recombination frequencies in different genotypes and resulting in inconsistent orders of loci. These are discussed in more detail in the following sections.

#### Multi-locus markers from segmental duplications

Ten and eight markers whose forward and reverse primer sequences, respectively, have shown strong similarities (*e*-value >0.01) with more than one linkage group. These markers point to a multi-locus feature arising from segmental duplications that may have rendered it difficult to score the correct allele, leading to differences in marker order in at least one of the linkage maps. It is known that the presence of duplicated genomic regions results in marker amplification problems, rendering them difficult to resolve on genotyping platforms, and resulting in errors in marker positioning [12]. For apple, structural variations in the genome, especially of segmental duplications, are common [19], [23]. Our evidence suggests that segmental duplications could be more abundantly present within certain regions of the apple genome. For example, nine markers are removed from LG 13, seven markers from LG 02, five from each of LGs 05 and 12, and none from LG 06. Also, of the 18 markers removed from the ‘Fiesta’ map, four markers are from LG 13. It is likely that there are more frequent structural variations on LGs 02, 05, 12, and 13. These results are in agreement with previous findings [19], wherein multiple markers with similarities to different chromosomes have been detected in LGs 02, 05, 12, and 13. It is also noteworthy to point out that different genotyping methods have been used in different SSR genotyping studies. For example, Silfverberg-Dilworth et al. [21] has resolved ^33^P-labeled PCR products on a 6% denaturing sequencing gel, while Han et al. [19] has used fluorescently labeled primers on capillary systems. Acrylamide gels and capillary systems have different powers of resolving differences in alleles, and this may have contributed to differences in scoring of alleles in different maps.

#### Structural variations among populations

In this study, structural variations among different populations may be responsible for observed differences in recombination rates that result in inconsistent marker order. This assumption could be supported if a genetic map from one population shows significant differences in marker order compared to other maps. Of a total of 60 markers removed, 11 were removed from the ‘M9’×‘R5’ map, which is the highest percentage of markers removed from any given map. The ‘M9’×‘R5’ map is derived from a cross between two wild apples, thus it is likely there may be structural differences in genomes of these two parents compared with parents of other mapping populations. Significant differences in recombination frequencies have been reported in several studies in other species; e.g., among three maps based on a double-haploid (DH) population of *Brassica napus* [32], among maize F2 populations [33], and among DH populations of *B. oleacera* [32]. A low correlation between a consensus map and a population-specific map, as well as a good correlation between the consensus map and other maps used for integration may be an indication of presence of structural variations among genomes of these populations [32], [34]. There will always be inconsistencies in marker order among multiple population maps due to differences in recombination frequencies caused by genomic structural variations between populations, in addition to differences in design, size, and marker density, as well as technical errors of genotyping [12]. In some species, rearrangements occur even over narrow evolutionary distances; for example, in maize, significant gene rearrangements between different lines of maize have been reported [31], [35]. This emphasizes that when developing a multiple population consensus map, genetic rearrangements among genomes of populations have to be taken into account for proper interpretation of marker order inconsistencies.

#### Primer design and primer sequences

Issues with primer design could cause poor amplification, ultimately influencing scoring, and contributing to differences in mapping positions between maps. There are 14 and eight markers whose forward and reverse primer sequences, respectively, do not show any significant similarities to the sequence of the ‘Golden Delicious’ (GD) apple genome sequence. Moreover, five markers do not show any significant sequence similarities for both forward and reverse primers, including NH029a, NH009b, and KA4b, originating from pear. Issues with these three pear markers strongly suggest that differences in marker order could be attributed to low sequence similarities between primer sequences of these markers and the GD genome sequence. These markers may have been difficult to use in genotyping (due to poor primer design leading to missing data and resulting in erroneous order of markers), and therefore contributing to misleading positioning of markers for at least some maps.

#### Mis-anchoring of the draft sequence of the apple genome

Presence of repetitive sequences has contributed to mis-assembly of some regions of the draft genome sequences [36]. According to Salzberg and Yorke [37], these mis-assemblies are common in regions where a genome is incorrectly re-arranged as well as in genomic regions wherein large segments of DNA sequences are simply deleted and surrounding sequences are compressed together. Findings in this study also point towards the possibility of mis-anchoring of the apple genome sequence due to problems in assembly of genomic regions with highly repetitive sequences. Primer sequences of six multi-locus markers (Hi07d12, CH01d03, CH02c02b, CH02a08, CH05g07, and CH02d10a) have mapped onto multiple linkage groups, showing similarities to the apple genome sequence for some chromosomes and corresponding to mapped linkage groups, but not to all corresponding linkage groups. The question as to why no sequence similarities are observed for all loci against corresponding chromosomes of the apple draft genome ought to be considered. It is likely that mis-assembly in such a region may have occurred, and that sequences from a locus on a linkage group lacking similarity may have been either assembled or anchored to the wrong chromosome. Moreover, due to the multi-locus feature of such a marker, it may have been difficult to score alleles for accurate genetic map construction as well. Therefore, these markers have resulted in differences in marker positions between maps, and MergeMap has subsequently removed them.

There are other indications of mis-assembly of some regions of the draft of the GD apple genome sequence, such as presence of markers Hi24f04, Hi02a03, Hi04a05, and Hi02c06 with sequence similarities to regions of a chromosome other than those of their corresponding linkage group. This is likely to be attributed to mis-anchoring of these genomic regions. Although the possibility that these markers are multi-allelic cannot be ruled out, it is expected that BLAST results should have at least revealed sequence similarities to the linkage group onto which they are mapped. For example, the marker CH03h03 maps only onto LG 13 in three maps, including the genetic map of Velasco et al. [23], but neither forward nor reverse primer sequences have shown any significant sequence similarities to chromosome 13. Instead, this marker shows that the reverse primer has similarity with a different chromosome. In another example, the marker Hi02c06 is also mapped onto LG 13 in two different genetic maps; however, sequences of both forward and reverse primers have not yielded any significant hits on chromosome 13. This finding also indicates that the sequence of the target region may have been mis-anchored to another duplicated region, on a different chromosome. However, the possibility that there could be a few markers with major order conflicts among maps, thereby displacing other markers and resulting in removal of multiple markers from the linkage group cannot be ruled out. Overall, these inconsistencies highlight some problems of assembly and anchoring of the GD genome sequences in regions where segmental duplications are present [19]. However, it should also be noted that some of the observed segmental duplications reported in the apple genome sequence may have resulted from assembly of the genome. It has been reported that genome assembly of diploid genomes with polymorphic regions in divergent chromosomes may be erroneously constructed, yielding an apparent recent segmental duplication [35].

## Materials and Methods

### Mapping population and DNA extraction

An F1 mapping population, derived from a cross between ‘Co-op 16’ and ‘Co-op 17’ as described by [19], was used. Young leaves were collected from 118 F1 seedlings and the two parents, all grafted onto Bud-9 apple rootstock and grown in a greenhouse at the University of Illinois, Urbana-Champaign. Leaves were freeze-dried in liquid nitrogen, and crushed into fine powder for genomic DNA extraction using the CTAB extraction method, with slight modifications as described previously [6]. DNA was quantified using a NanoDrop spectrophotometer (NanoDrop Technologies Inc., Wilmington, DE).

### SNP genotyping and scoring

A total of 250 ng genomic DNA, from each seedling and from both parents, was genotyped using the Illumina 1536 GoldenGate™ assay on the BeadStation system (Illumina Inc., San Diego, CA) at the W.M. Keck Center for Functional Genomics (University of Illinois at Urbana-Champaign), according to the manufacturer's protocol. Oligonucleotide pool assays (OPAs) for GoldenGate™ analysis consisted of 1536 SNPs, that included 1411 genic SNPs, previously described [6], along with an additional 125 genomic SNPs from the GD apple genome sequence [23].

The normalization procedure, removal of outliers, background correction, and scaling of raw hybridization intensity data were all carried out using the genotyping function in the BeadStudio package (Illumina, San Diego, CA) prior to genotype calling, as recommended by Illumina. Where needed, normalized intensity values, to one of three possible homozygous and heterozygous genotype clusters, were manually inspected and corrected. SNPs showing errors in segregation and with a GenCall (GC) score ≥0.25, based on an average GC scores for genotypes, were removed. SNPs with more than three clusters were deemed either erroneous or derived from paralog/homolog regions, and removed. Clean data were used to prepare a file of at most three genotypic classes.

### Construction of a new genetic map for ‘Co-op 16’ and ‘Co-op 17’

All genotypic data were checked for errors and for deviation from expected Mendelian segregation ratios using chi-square (x^2^) goodness-of-fit values. These data were combined with locus data files, previously developed [19] for physical and genetic map construction, and then used for linkage analysis using JoinMap version 4.0 [38]. Linkage groups were established using Haldane's mapping function with default calculation options and minimum LOD scores of 4.0. Each linkage group was individually checked for double recombinants. Markers showing a high number of double recombination events within a small genetic distance were re-scored, re-mapped, but removed from the dataset if the problem persisted. Moreover, those markers drastically disturbing orders of loci when compared to the integrated genetic map of Han et al. [19] were excluded from linkage analysis. After removal of outliers, the final map was constructed, again using the Haldane's mapping function with default calculation options and minimum LOD score of 4.0. The linkage map was graphically displayed using the MapChart program, v. 2.1 [39], according to the user's manual.

### Construction of a consensus genetic map and estimation of genome coverage

Map positions of SSRs and SNPs were obtained from the Genome Database for Rosaceae (GDR) website (http://www.rosaceae.org/) for four maps, including a map for each of ‘Fiesta’ and ‘Discovery’ [21], an integrated map based on an F1 pedigree of ‘Malling 9’ and ‘Robusta 5’ (M9×R5) [22], and an integrated map based on six F1 mapping populations [23]. Markers from each of the published maps, along with the newly constructed map for ‘Co-op 16’ and ‘Co-op 17’ were split into corresponding linkage groups. As a result, each linkage group had five individual maps. Minor variations in names of markers common across linkage groups were adjusted to ensure better integration, as the MergeMap algorithm utilizes names in common for anchoring linkage groups. Maps of each of the 17 linkage groups were given equal weight (weight = 1.0) to construct a consensus apple genetic map using MergeMap v1.2 [14]. In the final consensus map, hereafter referred to as the consensus apple genetic map, MergeMap removed markers showing conflicting positions across different maps of a linkage group. As the MergeMap inflated genetic distances between markers, this also inflated the length of the consensus genetic map. The length of each linkage group was averaged across different maps, and used to calculate a scaling factor.

The consensus apple genetic map was used to estimate genome coverage, calculated by averaging linkage map lengths and estimated using the method of Fishman et al. [25] and method 4 of Chakravarti et al. [26]. With Fishman et al.'s [25] methodology, average spacing of markers is doubled and then added to lengths of each linkage group; whereas, method 4 of Chakravarti et al. [26] expands each linkage group by (m+1)/(m−1), wherein m is the number of loci mapped.

### Identification of genomic regions with conflicts

Markers removed due to conflicts in map positions across different maps of a linkage group were investigated to determine causes of conflict. Both forward and reverse primer sequences of 45 SSR markers as well as sequences of 13 SNPs were retrieved from the public domain, and a BLASTn [40] search was performed for each sequence against an Apple Genome V1.0 contig dataset available at the GDR website. Default options were used with BLASTn 2.2.18 [40] along with filtering at low complexity, wherein ‘Expect’ is set at 10 and substitution matrix as BLOSUM-62 [41]. BLAST searches returned top 10 hits, and all hits with less than 80% overlap were removed first, and then all remaining hits were sorted based on e-values and hits. Those hits with *e*-values of less than 0.01 were also removed.

## Supporting Information

Figure S1 Conflicts in marker order among ‘Fiesta’ and ‘Discovery’ maps [21], M9×R5 map [22], an integrated map based on six populations [23], and our newly constructed consensus map, identified by MergeMap [14]. Each map is represented as a track, designated as file_0, file_1, file_2, file_3, and file_4 for the ‘Co-op 16’ and ‘Co-op 17’ map, ‘Discovery’ map [21], ‘Fiesta’ map [21], ‘M9’×‘R5’ map [22], and the integrated map [23], respectively. For LGs 03 and 14, track numbers are designated as file_0 up to file_5 as the linkage groups for the ‘Discovery’ map [21] are split into two, a and b. Thus, the tracks are designated as file_0, file_1, file_2, file_3, file_4, and file_5 for the ‘Co-op 16’ and ‘Co-op’ 17 map, ‘Discovery’ map A (top of original map [21], ‘Discovery’ map B (bottom of original map [21]), ‘Fiesta’ map [21], ‘M9’×‘R5’ map [22], and the integrated map [23], respectively. Each oval shape represents a single bin of markers, while the numbers between marker bins correspond to observed recombination frequencies. In the event an oval contains more than a single SNP, this indicates that there is no evidence of recombination in any mapping population between these markers.
(PDF)

Figure S2 Consensus map of apple showing the linear marker order after solving the conflicts in marker order among “Fiesta” and “Discovery” maps [21], M9×R5 map [22], an integrated map based on six populations [23] and our newly constructed map.
(PDF)

Table S1 A list of 1536 SNPs from GoldenGate™ OPA developed by Khan et al. (2012), along with their ≥60 bp flanking sequences, designability score, and rank. SNPs with 50% GenCall (GC)<0.25 were removed from further processing. Heterozygosity excess, missing data (Call Frequency), minor allele frequency, and 50% GC scores for all 1536 SNPs are provided. In addition, linkage group, map position (cM), segregation type, goodness of fit value (X2), and significance test for deviation from expected frequencies are also provided for those SNPs that were mapped onto the integrated map. Note: SNPs having scores >0.25 for 50% GC that were not mapped were either monomorphic, had excess missing data, or resulted in problems while establishing linkage groups due to some artifacts. Significance levels used for goodness of fit tests were: *:0.1 **:0.05 ***:0.01 ****:0.005 *****:0.001 ******:0.0005 *******:0.0001.
(XLSX)
