# Supplementary material for: A Multi-Population Consensus Genetic Map Reveals Inconsistent Marker Order among Maps Likely Attributed to Structural Variations in the Apple Genome
Source: PLoS One. 2012 Nov 5;7(11):e47864. doi: 10.1371/journal.pone.0047864 (PMC3489900; doi:10.1371/journal.pone.0047864)

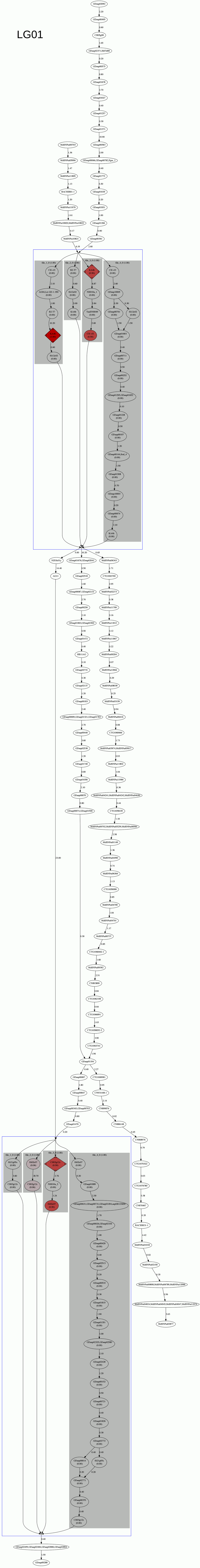
LG01

LG02

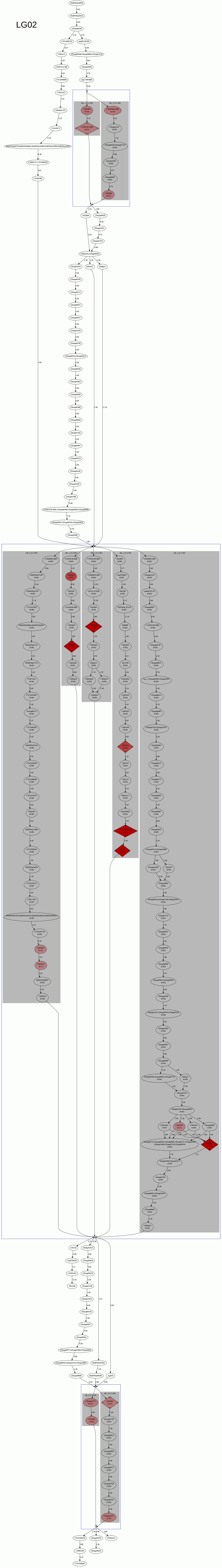

LG03

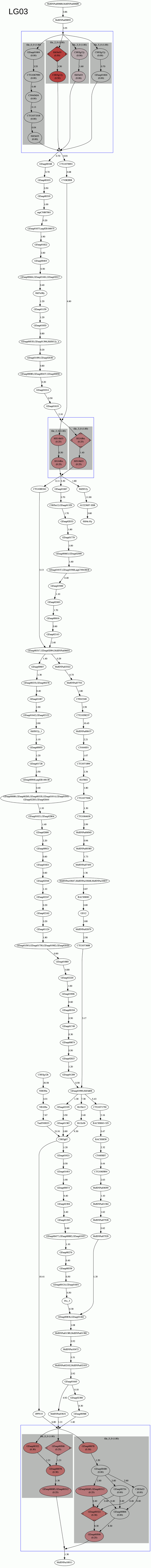

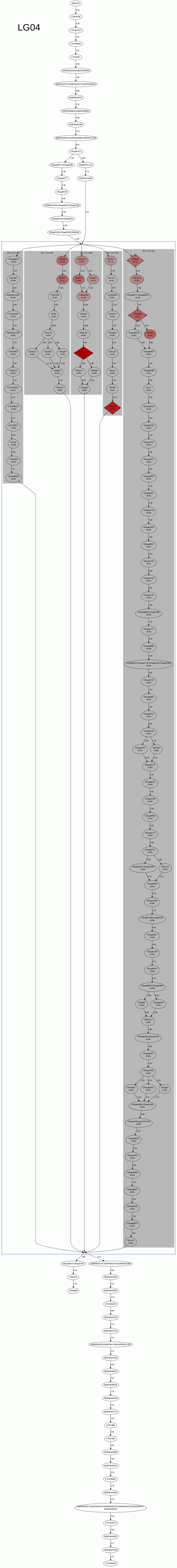
LG04

LG05

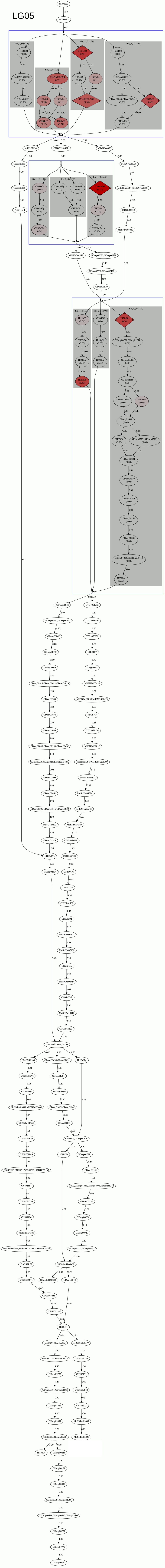

LG06

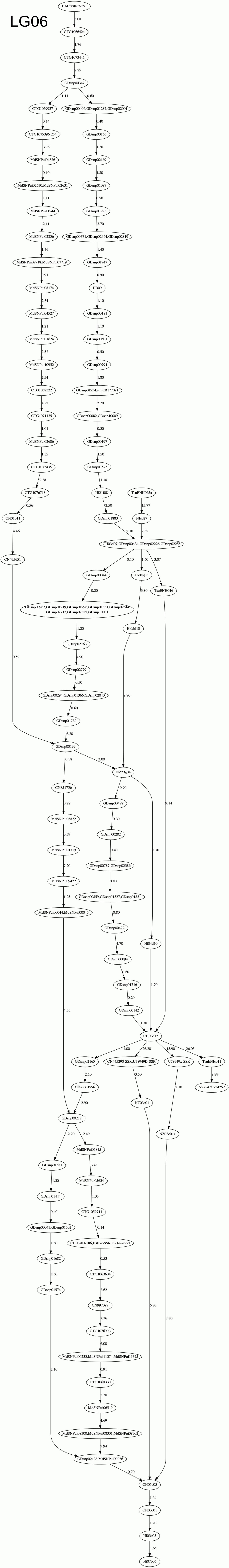

LG07

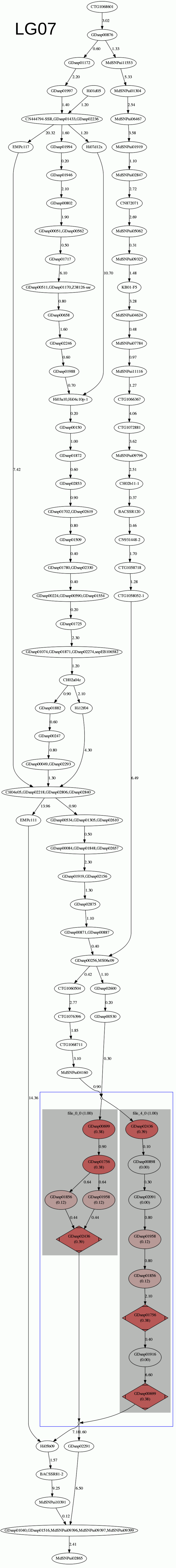

CTG1068601
GDsnp00876
GDsnp01172
MdSNPui11553
GDsnp01997
Hi01d05
MdSNPui01304
CN444794-SSR,GDsnp01433,GDsnp02236
MdSNPui06467
EMPc117
GDsnp01994
Hi07d12x
MdSNPui01919
GDsnp01946
MdSNPui02847
GDsnp00802
CN872071
GDsnp00051,GDsnp00562
MdSNPui05062
GDsnp01717
MdSNPui09322
GDsnp00511,GDsnp01170,Z38126-ssr
KB01-F5
GDsnp00658
MdSNPui04624
GDsnp02246
MdSNPui07784
GDsnp01988
MdSNPui11116
Hi03a10,Hi04c10p-1
CTG1066367
GDsnp00150
CTG1072881
GDsnp01872
MdSNPui09796
GDsnp02853
CH02b11-1
GDsnp01702,GDsnp02619
BACSSR120
GDsnp01509
CN931448-2
GDsnp01780,GDsnp02330
CTG1058718
GDsnp00224,GDsnp00590,GDsnp01554
CTG1058052-1
GDsnp01725
GDsnp01074,GDsnp01871,GDsnp02274,snpEB106583
CH02a04z
GDsnp01882
Hi12f04
GDsnp00247
GDsnp00049,GDsnp02293
CH04e05,GDsnp02218,GDsnp02806,GDsnp02840
EMPc111
GDsnp00534,GDsnp01305,GDsnp02610
GDsnp00084,GDsnp01848,GDsnp02657
GDsnp01919,GDsnp02156
GDsnp02875
GDsnp00871,GDsnp00887
GDsnp00256,MS06c09
CTG1060504
GDsnp02600
CTG1076396
GDsnp00530
CTG1068711
MdSNPui04160
file_0_0 (1.00)
file_4_0 (1.00)
GDsnp00699 (0.38)
GDsnp02436 (0.39)
GDsnp01756 (0.38)
GDsnp00898 (0.00)
GDsnp01856 (0.12)
GDsnp01958 (0.12)
GDsnp02091 (0.00)
GDsnp02436 (0.39)
GDsnp01958 (0.12)
GDsnp01856 (0.12)
GDsnp01756 (0.38)
GDsnp01916 (0.00)
GDsnp00699 (0.38)
Hi05b09
GDsnp02291
BACSSR81-2
MdSNPui10391
GDsnp01040,GDsnp01516,MdSNPui09396,MdSNPui09397,MdSNPui09399
MdSNPui02865

LG08

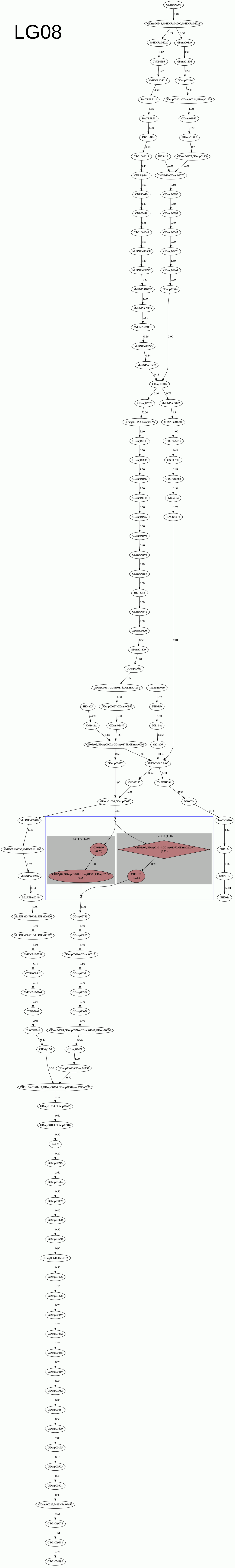

LG09

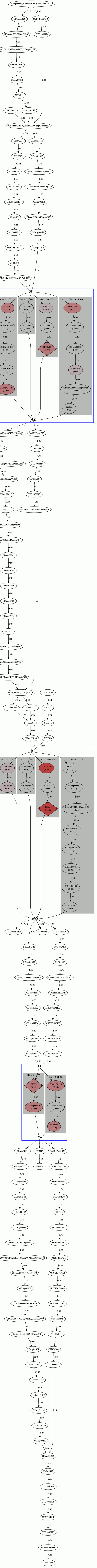

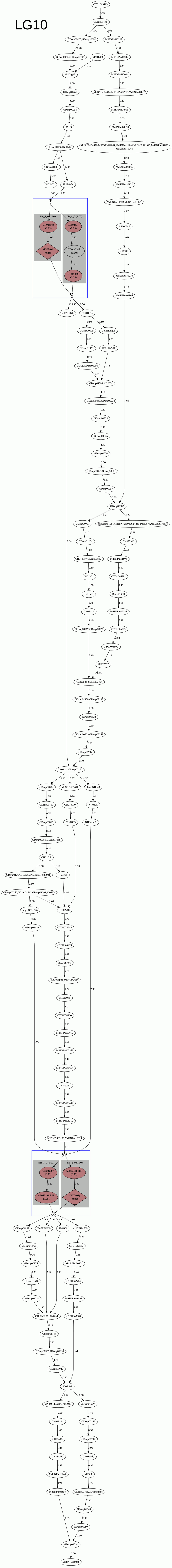
LG10

LG11

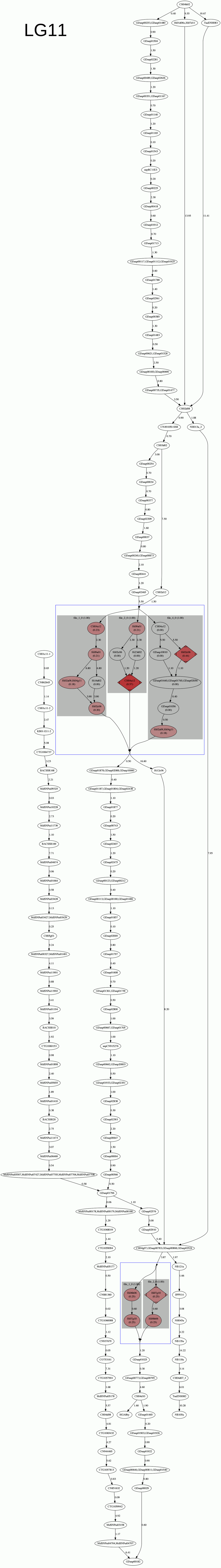

LG12

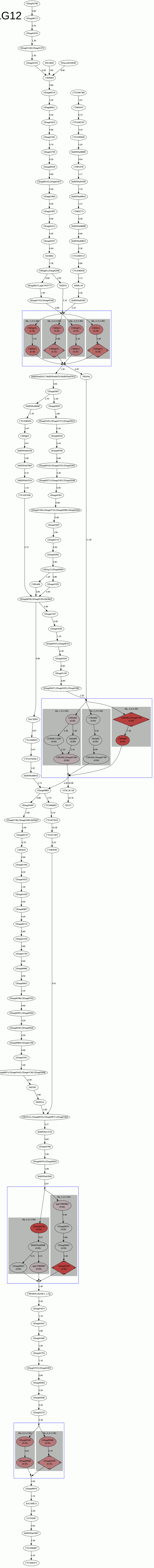

LG13

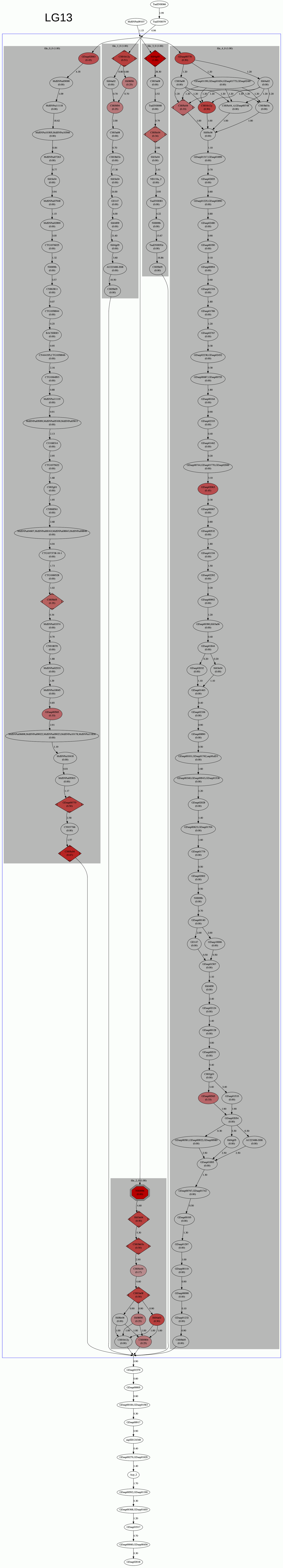

LG14

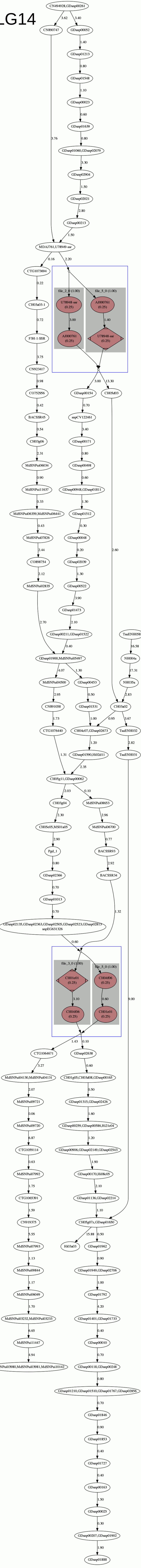

LG15

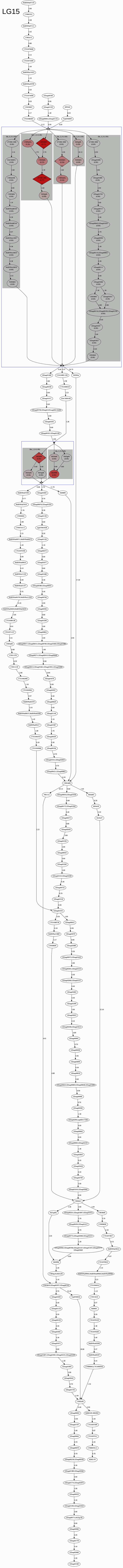

LG16

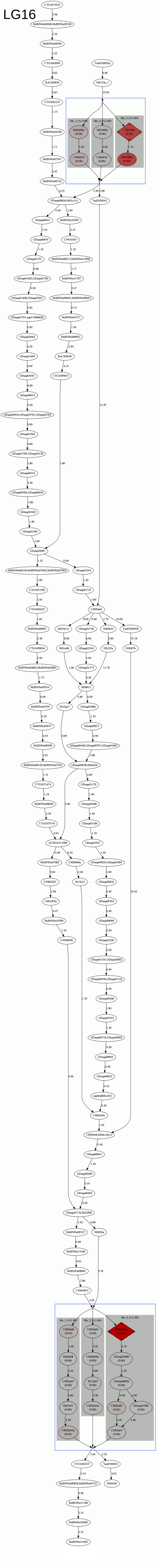

LG17

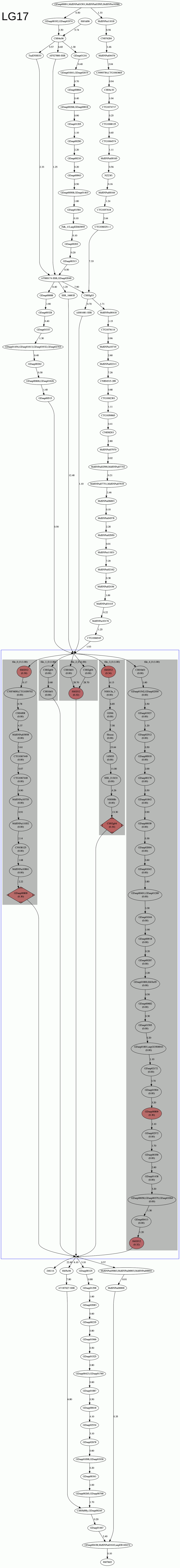

Supplement: Figure S1 — Conflicts in marker order among ‘Fiesta’ and ‘Discovery’ maps [21], M9×R5 map [22], an integrated map based on six populations [23], and our newly constructed consensus map, identified by MergeMap [14]. Each map is represented as a track, designated as file_0, file_1, file_2, file_3, and file_4 for the ‘Co-op 16’ and ‘Co-op 17’ map, ‘Discovery’ map [21], ‘Fiesta’ map [21], ‘M9’×‘R5’ map [22], and the integrated map [23], respectively. For LGs 03 and 14, track numbers are designated as file_0 up to file_5 as the linkage groups for the ‘Discovery’ map [21] are split into two, a and b. Thus, the tracks are designated as file_0, file_1, file_2, file_3, file_4, and file_5 for the ‘Co-op 16’ and ‘Co-op’ 17 map, ‘Discovery’ map A (top of original map [21], ‘Discovery’ map B (bottom of original map [21]), ‘Fiesta’ map [21], ‘M9’×‘R5’ map [22], and the integrated map [23], respectively. Each oval shape represents a single bin of markers, while the numbers between marker bins correspond to observed recombination frequencies. In the event an oval contains more than a single SNP, this indicates that there is no evidence of recombination in any mapping population between these markers. (PDF) [file pone.0047864.s001.pdf]
