# Supplementary material for: A Multi-Population Consensus Genetic Map Reveals Inconsistent Marker Order among Maps Likely Attributed to Structural Variations in the Apple Genome
Source: PLoS One. 2012 Nov 5;7(11):e47864. doi: 10.1371/journal.pone.0047864 (PMC3489900; doi:10.1371/journal.pone.0047864)
Supplement: Figure S2 — Consensus map of apple showing the linear marker order after solving the conflicts in marker order among “Fiesta” and “Discovery” maps [21], M9×R5 map [22], an integrated map based on six populations [23] and our newly constructed map. (PDF) [file pone.0047864.s002.pdf]

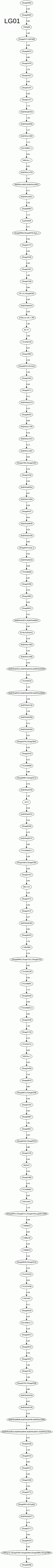
LG01

LG02

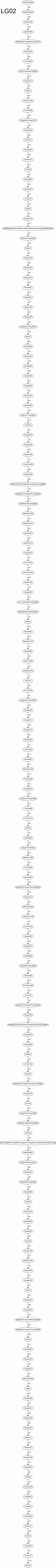
MdSNPui02620
0.03
MdSNPui02623
0.80
GDsnp00348
0.70
snpEB156780
0.50
GDsnp00408,GDsnp00856,GDsnp01134
0.50
GDsnp02026
0.64
CTG1068152
0.09
CN581493-SSR,snpCO903605
0.20
GDsnp01557
0.35
CH04e12
0.25
CN907414-200
0.39
CTG1066000
0.04
GDsnp00446,GDsnp01735
0.60
GDsnp01565
0.36
CN874327
0.35
GDsnp00959
0.80
CH04f04-675
0.01
CH02f06
1.80
GDsnp00035
0.30
GDsnp01641
0.38
CO416273
0.23
Hi22d06
0.11
GDsnp01476
0.44
MdSNPui00278,MdSNPui06961,MdSNPui10058,MdSNPui10288,MdSNPui10552
0.97
CH02c02a,GDsnp00203
0.50
Hi08g12
0.17
GDsnp01945
2.20
GDsnp00799
0.40
GDsnp00112
0.20
GDsnp00927
0.30
GDsnp00077
0.30
GDsnp01105
0.50
GDsnp00158
0.30
GDsnp00243,GDsnp00412
0.08
Hi02a07
0.09
GDsnp00228
1.40
GDsnp01663
0.50
GDsnp00850
0.50
GDsnp00308
0.30
GDsnp00095
0.30
GDsnp01162
0.30
GDsnp00497
1.06
CN863717,CTG1060102
0.67
CN495362
0.27
GDsnp00333
2.40
GDsnp00146
0.90
GDsnp01549
1.81
CN493139-SSR
0.90
GDsnp01498
0.60
CN493139-SSR1,GDsnp00366,GDsnp00504,GDsnp00505
1.10
GDsnp00407,GDsnp00549,GDsnp02055
0.10
AT000400-SSR,GDsnp00289
0.06
MdSNPui07248
0.20
MdSNPui07247
0.30
GDsnp01223
0.50
snpEB155133
0.60
GDsnp00991
0.47
CTG1073027
0.03
CN493139-SSR2
0.60
GDsnp00159
0.10
GDsnp00870
0.70
Aco_1,GDsnp00365,GDsnp01005
0.71
MdSNPui02046,MdSNPui02047
1.51
Hi07d12
0.33
GDsnp00614
0.26
MdSNPui07121
0.45
GDsnp00985
0.38
AJ251116-SSR
0.22
MdSNPui07123
1.41
CN871441
0.65
CTG1057807
0.41
GDsnp01552,GDsnp01553
0.20
GDsnp00483
0.80
GDsnp00214
1.80
GDsnp00174
0.70
GDsnp00002
1.17
CTG1062205
0.21
GDsnp00987
0.05
MdSNPui07416
0.76
CTG1062309
0.01
GDsnp00222
0.70
GDsnp02144,GDsnp02686
0.32
CTG1058066
1.99
CTG1074975
2.48
Hi24f04
2.77
TsuENH062
1.83
GDsnp01309,NH033b
0.94
MdSNPui11865
0.19
CTG1065429
0.28
GDsnp10004
0.30
GDsnp00425,GDsnp01758,GDsnp02812
0.50
GDsnp01743
0.24
MdSNPui01904
0.39
CH01K08_SCAR
1.18
CTG1070214
1.83
GDsnp02651
0.20
GDsnp00979
1.80
GDsnp00828
0.10
GDsnp00633,GDsnp00934
0.40
GDsnp01840
0.20
CN444636-SSR
0.02
CN917349
1.45
GDsnp01181,GDsnp02076,GDsnp02434
0.20
GDsnp01090
0.21
MdSNPui04342,MdSNPui04343,MdSNPui08420,MdSNPui08421
0.78
GDsnp02831
0.30
GDsnp00569
1.29
Hi08f05
0.50
CTG1057762
0.29
CH03d10
0.70
GDsnp00309,GDsnp00833,GDsnp01712,Hi05g12
1.60
GDsnp02155
0.77
BGT23b
0.18
GDsnp01935,GDsnp02093
1.90
CH02a04y,CH02c06,GDsnp00092
0.24
CH02b10
1.00
GDsnp00119,GDsnp00545,GDsnp00992,GDsnp01377,GDsnp01653,GDsnp01658,GDsnp02376,GDsnp02876
0.54
MdSNPui09991
0.56
GDsnp01829,GDsnp02271
0.90
GDsnp01251
0.50
GDsnp00022,GDsnp02449
0.30
GDsnp00655
1.30
CH05e03
0.30
GDsnp02223
1.80
GDsnp00629
0.50
GDsnp00478
0.88
CH01c06
1.58
GDsnp01349
0.64
MdSNPui07901
1.20
GDsnp01934
0.40
GDsnp00120
0.57
NH012a
0.50
GDsnp00267
0.50
GDsnp00624
0.40
GDsnp00973,GDsnp01868,GDsnp02061
0.80
GDsnp00019,GDsnp01532,GDsnp10007
0.08
NH212a
1.30
GDsnp00669
0.40
GDsnp01322
1.00
GDsnp00914
0.44
MdSNPui09939
0.53
argh46
0.44
GDsnp00995
0.40
GDsnp00471
0.90
GDsnp01938
0.50
GDsnp00167
0.14
NH010a_2
0.01
GDsnp01577
0.50
GDsnp02535
0.20
GDsnp00242
2.06
CH03d01
2.35
CTG1066318
0.59
TsuENH001
0.30
CN895349
0.10
CTG1065987
0.14
NZ03c01z
14.67
GD126
4.86
TsuENH045
3.11
CN581453
11.54
NB124b

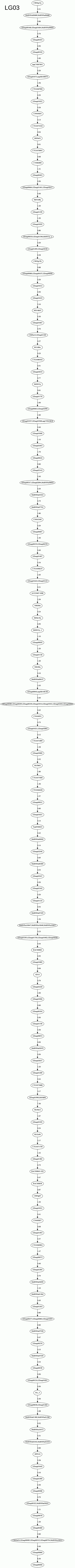
LG03
CH03g12y
0.31
MdSNPui00688,MdSNPui00689
0.86
GDsnp00168,GDsnp01604,MdSNPui09855
0.70
GDsnp00103
0.50
GDsnp00319
1.00
snpCN897901
0.20
GDsnp01073,snpEB106975
1.56
CTG1067990
0.29
GDsnp01822
0.26
CN945654
0.13
CTG1073318
0.04
Hi03e03
0.01
CTG1076901
0.68
CV082898
1.31
GDsnp00403
0.50
GDsnp00664,GDsnp01461,GDsnp02017
0.40
Hi07e08y
1.20
GDsnp01159
1.20
GDsnp01955
0.80
GDsnp00535,GDsnp01396,Hi05f12y_a
0.20
GDsnp01499,GDsnp02030
0.28
CH03g12b
0.88
GDsnp00080,GDsnp00437,GDsnp00950
0.20
GDsnp01014
0.50
GDsnp01633
2.10
MS14h03
0.30
GDsnp01667
0.70
CH05a12,GDsnp01329
0.27
HGA8bx
0.25
CTG1069342
0.51
GDsnp02033
0.77
Hi05f12y
0.61
GDsnp01779
1.00
GDsnp00663,GDsnp02599
1.60
GDsnp01937,GDsnp01968,snpCN943818
0.40
GDsnp01969
1.10
GDsnp02463
1.70
GDsnp00616
0.60
GDsnp02143
2.40
GDsnp00317,GDsnp02094,MdSNPui06652
0.29
MdSNPui03322
0.75
MdSNPui07701
1.80
CN943340
0.29
GDsnp00607
1.30
GDsnp00233,GDsnp00278
0.40
GDsnp01487
0.34
CTG1058237
1.65
GDsnp01642,GDsnp02122
0.21
AU223657-SSR
2.39
NH030a
0.24
Hi04c10y
0.59
Hi05f12y_1
1.10
GDsnp00955
1.20
GDsnp01728
1.20
NB109a
0.19
MdSNPui08437
1.63
GDsnp00940,snpEB148130
0.40
GDsnp00089,GDsnp00295,GDsnp00335,GDsnp01914,GDsnp01941,GDsnp02283,GDsnp02644
0.42
CN444051
0.75
GDsnp01021,GDsnp02804
0.12
CTG1071899
1.28
GDsnp02690
0.32
Hi15b02
1.80
CTG1077006
1.30
CTG1064630
1.57
GDsnp00921
0.60
GDsnp01821
0.14
TsuENH023
0.42
MdSNPui06565
0.14
GDsnp02546
0.65
MdSNPui00385
0.53
GDsnp02247
0.20
GDsnp02162
0.20
GDsnp01124
0.54
MdSNPui07455
1.36
MdSNPui10647,MdSNPui10648,MdSNPui10837
0.23
GDsnp01593,GDsnp01750,GDsnp01982,GDsnp02020
0.44
BACSSR82
0.55
GDsnp01885
0.06
GD12
0.79
GDsnp02220
1.00
GDsnp01906
0.60
GDsnp00194
0.50
GDsnp01749
0.30
GDsnp00874
0.09
MdSNPui02679
0.59
GDsnp02027
1.20
GDsnp01209
0.44
CTG1073688
3.17
GDsnp01990,Hi03d06
7.30
Hi15h12
1.67
GDsnp02195
0.24
Hi12c06
2.17
CTG1071750
1.42
GDsnp01382
0.75
BACSSR63-325
0.47
BACSSR58
0.87
CH03g07
1.20
GDsnp01921
0.41
CN495857
0.08
GDsnp01493
0.37
CTG1065894
1.47
GDsnp00073
0.40
GDsnp02304
0.79
MdSNPui04059
1.10
MdSNPui01364
0.45
GDsnp01263
0.60
GDsnp00477,GDsnp00882,GDsnp01957
0.60
MdSNPui07528
0.47
GDsnp00276
0.15
MdSNPui07529
0.24
GDsnp00539
0.50
GDsnp00124,GDsnp01851
0.50
Prx_3
0.30
GDsnp00836,GDsnp01482
1.08
MdSNPui01389,MdSNPui01390
0.52
MdSNPui10472
0.31
MdSNPui02102,MdSNPui02103
2.00
IPPN15
0.36
GDsnp01645
0.10
GDsnp02300
0.30
GDsnp00588
0.79
GDsnp00322,MdSNPui03631
1.21
GDsnp00078
1.20
GDsnp00480
2.60
CH03e03,GDsnp00085,GDsnp00312,GDsnp00754,MdSNPui10011
0.40
GDsnp00866
8.30
GDsnp00506

LG04

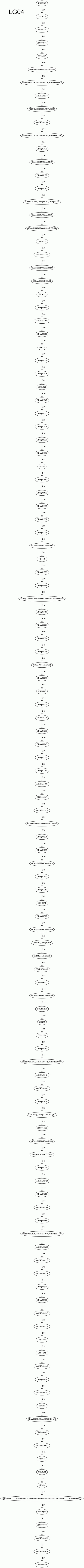
KB02-D2
0.50
CN870258
0.38
CTG1074157
1.61
CTG1069661
1.67
CN936557
1.80
MdSNPui02296,MdSNPui02299
1.85
MdSNPui04778,MdSNPui04779,MdSNPui05013
0.89
MdSNPui05347
3.70
MdSNPui06825,MdSNPui06826
0.46
MdSNPui01586
0.74
MdSNPui00201,MdSNPui06868,MdSNPui11268
0.01
GDsnp01471
0.30
GDsnp00523,GDsnp02189
0.30
GDsnp00177
0.30
GDsnp00160
0.50
AT000420-SSR,GDsnp00302,GDsnp01294
0.50
GDsnp00136,GDsnp00431
0.50
GDsnp01485,GDsnp02490,Hi08e04a
0.30
CH01b12x
0.67
MdSNPui11145
0.99
GDsnp00127,GDsnp00283
0.50
GDsnp00232,Hi08e04
3.50
Hi23d11
0.80
GDsnp00681
0.66
MdSNPui11082
0.40
GDsnp00388
0.20
Sod_3
0.30
GDsnp00226
0.40
GDsnp01026
0.05
CH02c02b
1.10
GDsnp01567
0.30
GDsnp00375
0.30
GDsnp02025
1.20
GDsnp00221
0.30
GDsnp01336
1.42
MSS6
1.46
GDsnp01965
1.30
GDsnp00619
0.20
GDsnp01195
0.60
GDsnp01558
0.90
GDsnp01236
1.40
GDsnp00686,GDsnp01006
0.45
NB141b
0.54
GDsnp01773
0.20
GDsnp00889
1.80
GDsnp00277,GDsnp01139,GDsnp01581,GDsnp02289
0.30
GDsnp01481
1.70
GDsnp00881
1.60
GDsnp00324
0.40
GDsnp00148
1.50
GDsnp02764,Hi07b02
0.30
GDsnp02277
1.61
CH01d07
0.64
GDsnp00321
1.10
TsuENH004
0.24
GDsnp01390
1.50
GDsnp00063
0.30
GDsnp01777
1.00
GDsnp02751
0.38
MdSNPui11991
0.46
CTG1064359
2.29
MdSNPui11939
0.24
GDsnp01292,GDsnp02296,Hi04c10x
0.70
GDsnp00928
0.70
GDsnp01095
1.10
GDsnp01766,GDsnp01929
0.60
GDsnp02817
4.20
GDsnp01353
0.07
CH01b09b
0.59
GDsnp00727
0.70
GDsnp00042,GDsnp01886
0.60
CH05d02,GDsnp02646
0.20
CH02h11a,Hi23g08
1.44
CTG1076206-1
3.24
CTG1060371
0.44
GDsnp00364,GDsnp02150
0.22
BACSSR14
0.46
GD162
0.99
CN993394
1.27
GDsnp00363
0.11
MdSNPui07147,MdSNPui07149,MdSNPui07398
0.05
MdSNPui03454
0.45
MdSNPui03643
0.68
GDsnp01928
0.20
CH01d03y,GDsnp02263,Hi23g02
2.58
CTG1064355
0.40
GDsnp01860,GDsnp01940
0.30
GDsnp01656,snpCO576145
0.10
GDsnp00705
0.18
MdSNPui02750
0.13
GDsnp01628
0.24
MdSNPui07106
0.27
GDsnp00966
0.71
MdSNPui02526,MdSNPui11046,MdSNPui11198
0.15
MdSNPui02528
0.09
MdSNPui06537
0.02
MdSNPui06536
0.11
GDsnp00904
1.50
GDsnp00768
0.17
MdSNPui06349
0.25
MdSNPui01714
1.04
CN911884
0.39
CN914285
0.92
MdSNPui04662
0.06
GDsnp00929
1.84
MdSNPui06347
1.28
Hi08h03
2.40
GDsnp00237,GDsnp01597,Hi01e10
0.19
CTG1064642
4.76
MdSNPui10081
0.12
NH011a
1.71
CH04e02
0.47
NH209a
0.16
MdSNPui05272,MdSNPui05274,MdSNPui05275,MdSNPui05276,MdSNPui05277,MdSNPui05278
1.09
NZ05g08
1.25
CTG1068776
3.05
MdSNPui05022
0.17
MdSNPui01026
1.37
CTG1066149

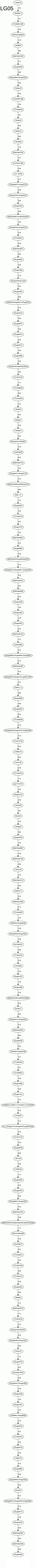
LG05
CH04c03
1.56
Hi05b09-2
4.47
CN496002-SSR
0.50
Hi02a03,Hi08a04
1.10
Hi08h08
3.89
MdSNPui07830
0.71
GDsnp00100
0.80
GDsnp00845,GDsnp02674
0.50
CH04e03
7.00
CN445599-SSR
1.66
CTG1064036
0.97
CH03a04
2.00
CH02b12y
3.00
CH02a08z
1.55
MdSNPui03709
0.47
AU223670-SSR
0.39
ATC_62636
0.12
GDsnp00075,GDsnp02729
0.40
GDsnp02332,GDsnp02427
0.50
GDsnp01539
0.08
MdSNPui00874,MdSNPui03551
1.05
GDsnp00739,GDsnp01751
0.24
CTG1065813
0.05
MdSNPui04012
0.09
GDsnp00382
0.20
GDsnp01859
2.10
GDsnp01020,Hi11a03
0.40
GDsnp01601
0.80
CH05f06,GDsnp02251,GDsnp02701
0.10
GDsnp02224
0.60
GDsnp00557
2.60
GDsnp00373
0.20
GDsnp00151
0.30
GDsnp00806
3.40
GDsnp01304,MdSNPui05443
3.05
CTG1061792
1.11
CTG1068636
0.95
CTG1076879
0.37
CH01b07
0.04
Hi20g01
0.39
CN996647
0.63
GDsnp01612,Hi04d02
0.36
TsuENH068
0.87
MdSNPui07414
1.02
GDsnp00231,GDsnp01722
0.12
MdSNPui02859,MdSNPui07413
0.09
KB01-A7
1.07
GDsnp00867
0.30
CTG1062470
0.53
GDsnp01478
1.82
MdSNPui09812
0.43
GDsnp00083
0.28
MdSNPui06759,MdSNPui06760
0.16
GDsnp00323,GDsnp00611,GDsnp01923
0.26
MdSNPui09121
0.87
MdSNPui08386
0.30
GDsnp01505
1.20
GDsnp01864
1.30
GDsnp01993
0.01
MdSNPui07242
1.27
TsuENH086
0.37
GDsnp00090,GDsnp00559,GDsnp00632
0.31
MdSNPui04587
0.16
GDsnp00978,GDsnp01515,snpEB134379
0.27
NH041a_4
0.60
GDsnp02809
0.80
GDsnp00461
0.04
CTG1069298
0.70
GDsnp00306,GDsnp01616,GDsnp01830
0.75
CTG1072784
0.03
CN890179
0.64
CN911587
0.38
CTG1061931
1.14
snpCO723472
0.20
GDsnp01319
0.30
CN870202
0.51
Hi21c08
2.10
CH04g09z
0.80
GDsnp02834
1.45
MdSNPui08897
0.39
MdSNPui07166
2.90
CN902156
1.63
MdSNPui04714
0.06
CH05e03-2
0.31
MdSNPui10918
0.73
CTG1064823
1.16
CH05e06,GDsnp00239
1.20
GDsnp00638,GDsnp01651
1.08
BACSSR164
0.68
CTG1061391
0.76
CN494669
0.44
GDsnp01781
1.10
GDsnp01896
1.55
MdSNPui03399,MdSNPui03400
0.55
Hi22a07y
2.90
GDsnp01671,GDsnp01942
0.23
MdSNPui08251
0.09
GDsnp00189
0.90
CH03a09,GDsnp01208
1.47
CTG1063619
0.61
CTG1058943
0.36
GDsnp01689
0.20
GDsnp01151
1.01
CN489916,CN896717,CO416051,CTG1059163
0.52
CN494587
0.13
Co_2,GDsnp01333,GDsnp01978,snpEB155242
0.35
CTG1074733
0.26
GDsnp00238
0.60
NB115b
0.28
CN899106
1.13
GDsnp00264
0.10
GDsnp00740
0.40
GDsnp00021,GDsnp01697
0.22
MdSNPui04331
0.08
MdSNPui02795,MdSNPui04269,MdSNPui04326
1.17
Hi01c04,Hi04a08
1.05
BACSSR75
0.07
CTG1065871
1.04
CTG1067456
0.66
CTG1061197
0.26
GDsnp00544
0.40
Hi09b04
1.31
MdSNPui08770
1.14
CTG1076729
0.33
GDsnp01620,Hi22f12
0.40
GDsnp00284,GDsnp01623
0.58
CN943151
1.21
GDsnp02732
0.25
NZmsEB155242
0.05
GDsnp00161,GDsnp01892
0.50
GDsnp01566
1.20
GDsnp02457
0.20
CH05h06z,GDsnp00900
0.10
GDsnp00244
0.32
CTG1063512
0.19
GDsnp00179
0.25
CN881672
0.32
GDsnp00895
0.40
GDsnp00054,GDsnp01956
0.64
Hi15e04
0.14
GDsnp00521,GDsnp00526,GDsnp01694
0.70
GDsnp00737
1.00
GDsnp01979
0.46
MdSNPui03697
0.09
MdSNPui06328
0.96
GDsnp00460

LG06

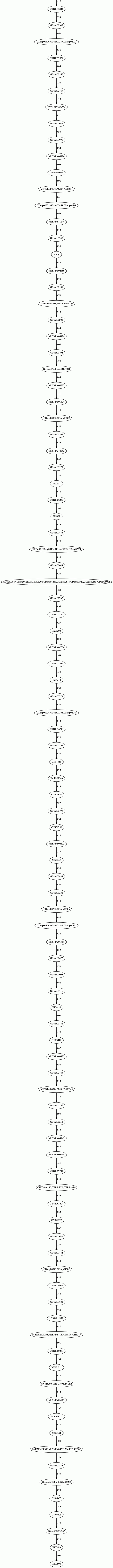

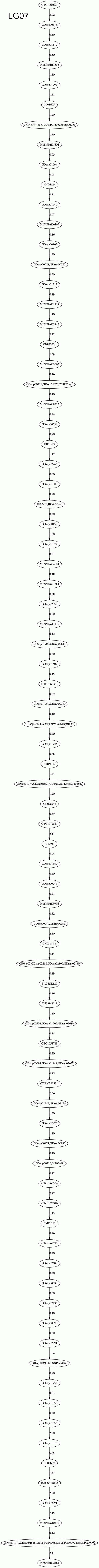
LG07
CTG1068601
3.02
GDsnp00876
0.60
GDsnp01172
0.50
MdSNPui11553
1.80
GDsnp01997
1.61
Hi01d05
1.20
CN444794-SSR,GDsnp01433,GDsnp02236
1.70
MdSNPui01304
0.03
GDsnp01994
0.08
Hi07d12x
0.11
GDsnp01946
2.07
MdSNPui06467
0.16
GDsnp00802
1.90
GDsnp00051,GDsnp00562
0.50
GDsnp01717
0.49
MdSNPui01919
1.10
MdSNPui02847
2.72
CN872071
2.69
MdSNPui05062
0.16
GDsnp00511,GDsnp01170,Z38126-ssr
0.10
MdSNPui09322
0.84
GDsnp00658
0.70
KB01-F5
1.12
GDsnp02246
0.60
GDsnp01988
0.70
Hi03a10,Hi04c10p-1
0.20
GDsnp00150
1.00
GDsnp01872
0.01
MdSNPui04624
0.48
MdSNPui07784
0.28
GDsnp02853
0.60
MdSNPui11116
0.12
GDsnp01702,GDsnp02619
0.80
GDsnp01509
0.15
CTG1066367
0.29
GDsnp01780,GDsnp02330
0.40
GDsnp00224,GDsnp00590,GDsnp01554
0.20
GDsnp01725
0.88
EMPc117
1.34
GDsnp01074,GDsnp01871,GDsnp02274,snpEB106582
1.20
CH02a04z
0.89
CTG1072881
2.17
Hi12f04
0.04
GDsnp01882
0.60
GDsnp00247
0.21
MdSNPui09796
0.82
GDsnp00049,GDsnp02293
2.60
CH02b11-1
0.14
CH04e05,GDsnp02218,GDsnp02806,GDsnp02840
0.19
BACSSR120
0.46
CN931448-2
1.40
GDsnp00534,GDsnp01305,GDsnp02610
0.14
CTG1058718
0.30
GDsnp00084,GDsnp01848,GDsnp02657
0.85
CTG1058052-1
2.06
GDsnp01919,GDsnp02156
1.30
GDsnp02875
1.10
GDsnp00871,GDsnp00887
0.40
GDsnp00256,MS06c09
0.42
CTG1060504
2.77
CTG1076396
1.15
EMPc111
0.76
CTG1068711
0.20
GDsnp02600
0.20
GDsnp00530
0.30
GDsnp02436
0.10
GDsnp00898
0.30
GDsnp02091
1.64
GDsnp00699,MdSNPui04160
0.90
GDsnp01756
0.64
GDsnp01958
0.80
GDsnp01856
2.50
GDsnp01916
5.65
Hi05b09
1.57
BACSSR81-2
2.00
GDsnp02291
7.15
MdSNPui10391
0.12
GDsnp01040,GDsnp01516,MdSNPui09396,MdSNPui09397,MdSNPui09399
2.41
MdSNPui02865

LG08

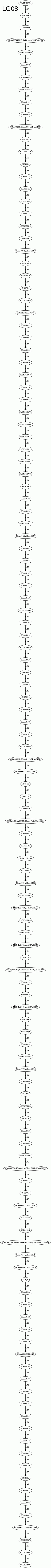

LG09

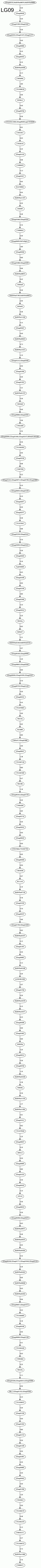

LG10

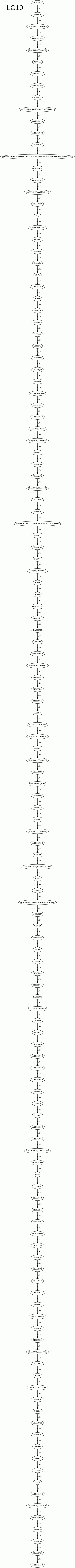

LG11

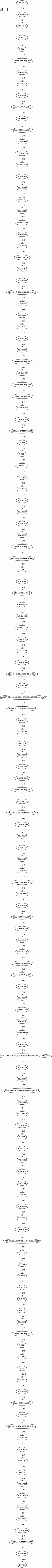
CH01c11-1
0.65
CN862645
1.14
CH01c11-2
2.47
KB01-E11-2
3.51
CH04h02
0.40
GDsnp00253,GDsnp01480
0.90
GDsnp01504
0.18
CTG1064737
1.22
GDsnp02281
1.30
GDsnp00489,GDsnp02626
0.69
BACSSR168
0.53
GDsnp00351,GDsnp01167
0.70
GDsnp01140
0.89
MdSNPui09325
0.03
MdSNPui10228
0.27
GDsnp01165
0.10
GDsnp01543
0.20
snpRC11E3
0.20
GDsnp00225
1.74
MdSNPui11739
0.48
GDsnp00418
0.60
GDsnp01912
0.90
Hi01d06x,Hi07d11
0.60
BACSSR169
0.41
GDsnp01713
1.30
GDsnp00117,GDsnp01112,GDsnp01925
0.60
GDsnp01788
1.09
TsuENH083
0.31
GDsnp02561
0.20
GDsnp00385
1.30
GDsnp01483
0.50
GDsnp00621,GDsnp01528
1.80
MdSNPui04674
0.89
GDsnp00185,GDsnp00690
0.80
GDsnp00735,GDsnp01477
1.61
MdSNPui01664
0.58
MdSNPui03428
0.13
MdSNPui03427,MdSNPui03429
0.25
CH05g01
3.94
CH02d08
0.90
CN491050-SSR
0.70
CH03d02
3.00
GDsnp00254
0.70
GDsnp00034
0.70
GDsnp00377
0.80
GDsnp02309
1.60
GDsnp00037
0.80
GDsnp00240,GDsnp00873
1.31
MdSNPui00327,MdSNPui01481
1.81
CH02d12
0.22
GDsnp00441
1.20
CH04a12,GDsnp02465
2.30
Hi09a01
0.72
MdSNPui11901
0.88
MdSNPui11902
2.69
NH013a_2
0.45
GDsnp10010
0.97
MdSNPui01194
0.08
GDsnp01640,GDsnp01765,GDsnp02056
0.40
GDsnp01056
0.50
GDsnp01878,GDsnp02088,GDsnp10000,Hi02a09,Hi04g11,Hi16d02
0.40
GDsnp01187,GDsnp01804,GDsnp02438
1.10
GDsnp01877
0.20
GDsnp00743
0.10
BACSSR10
1.16
GDsnp02407
1.20
GDsnp02475
0.00
Hi02c06,Hi23d02
0.12
GDsnp00123,GDsnp00212
0.23
CTG1060153
0.16
GDsnp00113,GDsnp00180,GDsnp01486
1.05
MdSNPui01898
0.13
GDsnp01857
0.10
GDsnp02005
0.80
GDsnp01797
0.40
GDsnp01608
0.70
GDsnp01361,GDsnp01739
0.43
MdSNPui05655
0.07
GDsnp02800
1.00
GDsnp00667,GDsnp01705
0.82
MdSNPui01410
0.38
BACSSR20
0.06
snpCN915276
1.10
GDsnp00662,GDsnp20003
0.50
GDsnp01933,GDsnp02351
1.00
GDsnp02838
0.30
GDsnp02393
0.29
MdSNPui11473
1.81
GDsnp00647
1.26
MdSNPui06469
0.08
GDsnp00004
0.46
MdSNPui05507,MdSNPui07427,MdSNPui07705,MdSNPui07706,MdSNPui07708
0.52
GDsnp00566
0.50
GDsnp01796
0.06
MdSNPui00178,MdSNPui00179,MdSNPui00180
1.29
CTG1068019
1.44
CTG1059094
2.10
MdSNPui05177
1.50
Hi12c06
1.61
CN881360
0.02
CTG1060088
1.12
CN937679
0.05
CO753161
0.91
GDsnp02576
3.00
GDsnp02910
0.87
CTG1057901
1.68
MdSNPui05178
0.71
CH04g07,GDsnp00783,GDsnp00868,GDsnp02524
1.97
NB121a
1.66
IPPN14
3.08
NH045a
4.22
NB135a
1.80
Hi06b06
0.60
Hi07g10
1.85
GDsnp01025
0.30
GDsnp00773,GDsnp00795
2.94
CH04d08
1.98
CH04d10
1.60
HGA8by
11.47
CTG1065432
0.02
GDsnp01465
0.20
GDsnp01903,GDsnp01926
0.90
GDsnp01922
0.90
GDsnp00646,GDsnp00811,GDsnp01540
0.80
GDsnp00029
6.75
NB118a
1.37
CN944465
0.65
CH04d07_f
4.98
CTG1057813
0.83
CN851632
0.09
CTG1059942
2.24
TsuENH082
1.01
MdSNPui03158
1.17
MdSNPui04704,MdSNPui04707
6.41
GDsnp00182
18.02
NB105a

LG12

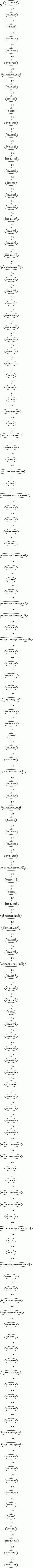
NZmsAB162040
3.60
GDsnp02789
0.45
MS14b04
0.15
GDsnp00173
0.70
GDsnp01070
1.08
CTG1067366
0.22
GDsnp01168,GDsnp01270
1.30
GDsnp01910
0.35
CN904191
0.02
CH05d04
0.32
CTG1063707
2.17
GDsnp00116
0.93
CTG1059629
1.45
MdSNPui08089
1.30
GDsnp00011
0.52
CN851978
0.02
GDsnp01615
0.60
GDsnp01391
0.04
MdSNPui01058
0.72
GDsnp01795
0.20
GDsnp00430
0.02
MdSNPui06943
1.54
GDsnp00145,GDsnp01837
0.40
GDsnp01690
0.50
GDsnp01907
0.25
CN892773
0.48
MdSNPui08088
0.69
MdSNPui00823
1.23
GDsnp02212
0.20
GDsnp02537
0.85
CTG1065713
0.17
NZ28f04
0.63
CTG1060429
1.12
KB08-A9
0.07
CH04g04,GDsnp02560
2.50
NH207a
0.75
GDsnp00272,snpCN937771
0.88
MdSNPui02487
0.08
CH05g07y
2.68
CH05d11,GDsnp01703,GDsnp02365
0.55
NB104a
0.73
MdSNPui02417,MdSNPui04570,MdSNPui07912
0.84
GDsnp02857
2.30
GDsnp02053
0.16
MdSNPui00983
1.35
CTG1060456
0.32
GDsnp01052,GDsnp01373,GDsnp02814
0.10
GDsnp00220
0.04
CH05g02
0.06
GDsnp00700
0.60
GDsnp00318,GDsnp02379,GDsnp02502
0.20
GDsnp00275,GDsnp01492,GDsnp02048
0.50
GDsnp01501
0.60
GDsnp01284,GDsnp01724,GDsnp02089,GDsnp02494
0.60
GDsnp01607
1.90
GDsnp02115
0.35
MdSNPui04438
0.15
GDsnp02582
0.90
CH01g12,GDsnp00599
0.69
MdSNPui07805
0.15
MdSNPui01524
0.06
CH01d09
0.45
GDsnp01555
0.68
CTG1057656
0.72
GDsnp00528,GDsnp02326,Hi03b03
1.00
GDsnp01357
0.50
GDsnp01029
1.10
GDsnp00193,GDsnp00314
0.29
BACSSR2
0.10
GDsnp01045
0.60
GDsnp01150
0.39
CTG1065053
0.19
GDsnp00457,GDsnp01053,GDsnp02085
0.82
CTG1076206-2
0.20
CH04d02
1.57
MdSNPui00015
0.09
CN496913-SSR,Hi02d05
1.50
CH01f02,GDsnp01798
4.40
GDsnp00883
0.80
GDsnp01995
1.00
GDsnp01769,GDsnp01984,Hi02b07
1.00
GDsnp00747
0.16
CTG1066003
0.19
CTG1072632
0.41
CH03c02
0.90
GDsnp01394
0.20
GDsnp01022
1.20
GDsnp01420
0.50
GDsnp00807
0.40
GDsnp00714
0.14
AT20_SCAR
0.69
GDsnp01426
0.80
GDsnp01759
0.90
GDsnp00685
0.50
GDsnp00641
2.10
GDsnp00386,GDsnp00762
0.60
GDsnp00391,GDsnp00464
1.66
CTG1071851
0.53
CV082939
0.96
GDsnp00362,GDsnp00620
0.50
GDsnp00840,GDsnp01180
0.60
GDsnp01051
1.00
GDsnp00974,GDsnp01610,GDsnp01760,GDsnp02008
0.40
Hi07f01
2.60
Hi05f12x
3.50
CH01b12z,GDsnp00334,GDsnp00937,GDsnp01662
0.27
MdSNPui11532
0.65
GDsnp01568
1.66
GDsnp00376,GDsnp00925
1.08
GDsnp02228,MdSNPui03582
0.97
MdSNPui00998
0.21
snpCN899883
0.40
GDsnp00251
0.80
GDsnp00653
2.40
CH01d03z,Hi23d11_1,Vg
0.40
GDsnp01874
1.10
GDsnp01647
1.80
GDsnp01665
1.90
GDsnp01793
1.10
GDsnp01523,GDsnp01855
0.90
GDsnp00296,GDsnp00338
0.20
GDsnp02848
0.20
GDsnp02152
0.02
GDsnp00068
1.80
GDsnp00915
4.34
BACSSR13
0.22
GD127
0.21
CO754587
0.02
MdSNPui01007
5.39
CTG1066897
0.34
CTG1064471

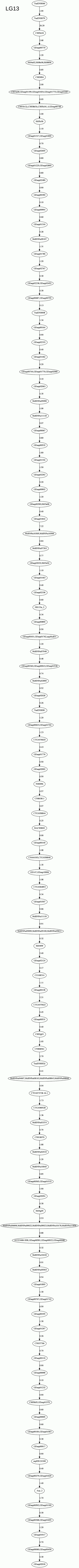
LG13
TsuENH066
1.99
TsuENH079
26.29
CH05c04
2.68
GDsnp00770
1.20
Hi04a02,Hi08e06,Hi08f06
0.85
CH05f04
2.00
CH03a08,GDsnp01199,GDsnp01654,GDsnp01775,GDsnp02487
0.95
CH01b12y,CH03h03z,CH05c04_4,GDsnp00748
1.00
Hi05c06
1.10
GDsnp01317,GDsnp01899
0.70
GDsnp02655
0.80
GDsnp01225,GDsnp02890
0.80
GDsnp02480
0.90
GDsnp00350
0.10
GDsnp00994
0.60
GDsnp01316
0.28
MdSNPui09107
1.31
GDsnp01786
1.20
GDsnp02767
0.30
GDsnp02238,GDsnp02452
0.30
GDsnp00687,GDsnp00755
0.13
TsuENH008
1.34
GDsnp00164
0.90
GDsnp02333
0.40
GDsnp01462
0.20
GDsnp00744,GDsnp01770,GDsnp02069
3.10
GDsnp02063
4.35
MdSNPui05098
1.09
MdSNPui11118
0.07
GDsnp00067
0.80
GDsnp00532
1.80
GDsnp01334
1.50
GDsnp02292
0.20
GDsnp00902
1.20
GDsnp00390,Hi03a06
0.40
GDsnp01844
7.22
MdSNPui10305,MdSNPui10566
0.84
MdSNPui07263
0.77
GDsnp02032,Hi03e04
1.10
GDsnp01463
0.40
GDsnp02336
0.60
NB133a_2
0.34
GDsnp00890
0.50
GDsnp00101,GDsnp01762,snpMalD1
1.20
MdSNPui07948
0.48
GDsnp00340,GDsnp00943,GDsnp01536
0.74
MdSNPui02890
0.52
GDsnp02028
0.26
TsuENH081
1.20
GDsnp00623,GDsnp01704
1.23
CTG1076025
0.43
GDsnp01776
0.50
GDsnp02002
0.50
NH009b
0.57
CN863811
0.07
CTG1058844
0.25
BACSSR83
0.00
GDsnp00140
1.08
CN444195,CTG1058846
1.35
GD147,GDsnp10006
1.08
CTG1064893
0.34
GDsnp01507
0.66
MdSNPui11119
0.91
MdSNPui05099,MdSNPui05100,MdSNPui05811
0.32
Hi04f09
2.40
GDsnp02124
0.17
CO168314
1.11
GDsnp00128
2.21
CTG1075622
0.45
GDsnp00531
5.40
CH02g01
1.95
CN868561
2.70
TsuENH052c
0.41
MdSNPui04607,MdSNPui08163,MdSNPui08843,MdSNPui08846
4.04
CTG1073738-16-1
1.73
CTG1069528
1.36
MdSNPui03374
0.79
CN918070
1.88
MdSNPui02533
1.29
MdSNPui10045
0.85
GDsnp00565,GDsnp01533
1.90
GDsnp02054
0.30
Hi04g05
2.53
MdSNPui06698,MdSNPui09022,MdSNPui09023,MdSNPui10178,MdSNPui11856
0.88
AU223486-SSR,GDsnp00581,GDsnp00922,GDsnp00980
0.32
MdSNPui10418
0.01
MdSNPui05503
0.54
GDsnp01895
1.30
GDsnp00767,GDsnp01742
0.50
GDsnp00195
1.30
GDsnp01297
0.26
CN937766
0.70
GDsnp00110
0.90
GDsnp00098
0.10
GDsnp01232
0.90
CH05h05,GDsnp01579
0.60
GDsnp00005
0.60
GDsnp00184,GDsnp01583
0.30
GDsnp00917
0.90
snpEB124348
0.40
GDsnp00279,GDsnp01629
1.40
Acp_2
1.70
GDsnp00592,GDsnp01190
0.30
GDsnp00368,GDsnp01655
1.20
GDsnp01917
0.70
GDsnp00060,GDsnp00456
0.30
GDsnp02018

LG14

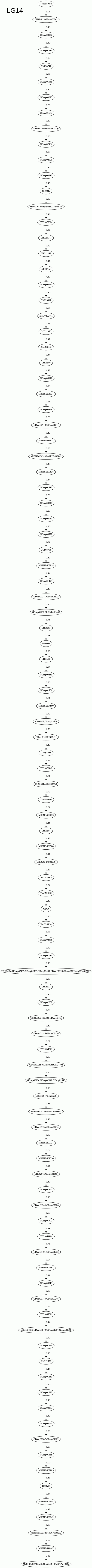
TsuENH058
3.05
CN494928,GDsnp00261
3.40
GDsnp00052
1.40
GDsnp01213
0.34
CN890747
0.38
GDsnp01548
1.10
GDsnp00023
0.60
GDsnp01639
0.80
GDsnp01060,GDsnp02079
3.30
GDsnp02904
1.50
GDsnp02021
2.80
GDsnp00213
0.13
NH004a
0.33
MDAJ761,U78949-ssr,U78948-ssr
0.16
CTG1073694
0.22
CH03a03-1
0.72
F3H-1-SSR
0.12
AJ000761
4.40
GDsnp00154
0.10
CN923417
0.55
snpCV122461
0.43
CO752956
0.42
BACSSR45
0.54
CH03g06
1.82
GDsnp00171
0.53
MdSNPui06034
0.21
GDsnp00498
0.60
GDsnp00948,GDsnp01811
0.12
MdSNPui11637
0.33
MdSNPui06359,MdSNPui06441
0.43
MdSNPui07826
0.34
GDsnp01512
0.30
GDsnp00048
0.20
GDsnp02039
1.30
GDsnp00522
0.37
CO898754
2.12
MdSNPui02835
1.14
GDsnp01473
2.10
GDsnp00211,GDsnp01522
0.40
GDsnp01900,MdSNPui05497
0.66
CH05d03
0.78
NH035a
2.83
CH03a02
0.64
GDsnp00453
0.50
GDsnp01531
0.01
MdSNPui04500
0.79
CH04c07,GDsnp02673
1.20
GDsnp01590,Hi02d11
1.17
CN891058
1.73
CTG1076440
1.31
CH05g11,GDsnp00062
0.99
TsuENH032
0.01
MdSNPui08653
1.15
CH03g04
1.85
MdSNPui06700
0.41
CH05e05,MS01a05
0.37
BACSSR93
0.31
TsuENH031
2.49
Pgd_1
0.70
BACSSR34
0.08
GDsnp02366
0.70
GDsnp01013
0.70
CH04f06,GDsnp02135,GDsnp02363,GDsnp02505,GDsnp02523,GDsnp02813,snpEG631326
0.60
CH01e01
0.10
GDsnp02638
0.60
CH01g05,CH03d08,GDsnp00165
0.50
GDsnp01315,GDsnp02426
0.02
CTG1064671
1.33
GDsnp00259,GDsnp00586,Hi21e04
1.20
GDsnp00906,GDsnp02149,GDsnp02543
1.90
GDsnp00170,Hi08c05
0.15
MdSNPui04130,MdSNPui04131
1.46
GDsnp01136,GDsnp02214
0.69
MdSNPui09721
0.06
MdSNPui09720
0.92
CH05g07z,GDsnp01650
0.50
GDsnp01962
0.90
GDsnp01949,GDsnp02706
1.00
GDsnp01792
3.58
CTG1059114
0.62
GDsnp01401,GDsnp01733
0.04
MdSNPui07992
0.41
GDsnp00010
0.70
GDsnp00118,GDsnp00248
0.64
CTG1065391
0.14
GDsnp01210,GDsnp01510,GDsnp01767,GDsnp02856
0.70
GDsnp01846
0.75
CN919375
0.15
GDsnp01853
0.40
GDsnp01727
0.40
GDsnp00163
1.50
GDsnp00025
0.30
GDsnp00207,GDsnp01902
1.90
GDsnp01888
1.00
MdSNPui07993
0.29
Hi03a03
0.84
MdSNPui09844
1.17
MdSNPui06049
1.70
MdSNPui03232,MdSNPui03233
6.65
MdSNPui11447
4.94
MdSNPui03980,MdSNPui03981,MdSNPui10142

LG15

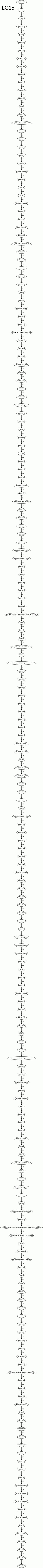

LG15
MdSNPui07110
2.00
CN862321
2.46
IPPN05
0.02
MdSNPui07111
2.42
CH02e12
0.80
CTG1076069
0.14
CTG1073209
1.49
MdSNPui11923
1.35
MdSNPui10759
0.77
GDsnp00349
0.50
GDsnp01325
0.50
TsuENH093
0.48
CTG1074058
0.03
CN939907
0.17
CTG1060287
0.12
GDsnp00694,GDsnp01131,Z71981-SSR
0.50
GDsnp01009
0.90
GDsnp01680
0.50
GDsnp01763
0.60
GDsnp00717
0.11
BACSSR51
1.68
GDsnp00642,GDsnp01634
0.70
GDsnp00550
0.31
CH02d10b
0.03
CH05a02z
0.97
GDsnp00115,GDsnp00842
0.30
GDsnp00111
0.90
GDsnp01666
1.30
CH03b06,GDsnp01814
1.19
MdSNPui08745
0.23
GDsnp00134,GDsnp00329,GDsnp01318
0.17
MdSNPui08744
0.38
GDsnp01047
0.30
MdSNPui11907
0.36
MdSNPui10633
0.28
MdSNPui10632
0.35
Hi02g06
0.44
GDsnp01827
0.40
GDsnp01469,NZ02b01
1.60
GDsnp00330
0.40
GDsnp01517
0.40
GDsnp00758,GDsnp01454,snpEB134400
0.24
CO414802-156
1.58
CTG1069215
1.13
GDsnp01551
0.60
GDsnp00191,GDsnp01185
0.44
BACSSR108
1.28
Hi03a06,Hi03g06
1.70
GDsnp01820
0.05
MdSNPui07494
0.59
GDsnp00076,GDsnp01254
0.19
MdSNPui07631
0.10
GDsnp01130
0.01
Hi03f09
0.58
snpDT001928
0.20
GDsnp01120
1.00
CN908484
0.03
GDsnp00274
0.60
GDsnp01077
0.60
GDsnp01488
0.30
GDsnp00389,GDsnp00831
0.47
CN907414-1
1.21
MdSNPui00571,MdSNPui00572
1.45
CTG1075196
0.59
MdSNPui00614
0.15
MdSNPui11155
0.27
GDsnp00796
0.23
MdSNPui01157
0.70
MdSNPui06378,MdSNPui11203
0.19
MdSNPui04818,MdSNPui04819
0.65
GDsnp00396
0.24
NH204a
0.59
GDsnp01206
0.49
CTG1069128
0.91
CTG1071157
0.11
GDsnp00894
1.60
GDsnp00017,GDsnp00031,GDsnp00785,GDsnp01660,GDsnp01989
0.09
Hi06f09
0.80
CH04a06
0.60
CN911379
0.35
GDsnp00271,GDsnp00543,GDsnp00640
0.00
CN911135
0.86
GDsnp00012,GDsnp01661,GDsnp01901,GDsnp02869
0.90
GDsnp01876
0.50
GDsnp00397
0.40
GDsnp00625
0.11
CTG1062468
0.36
GDsnp01146
0.87
CTG1063001
0.19
GDsnp01587
1.70
GDsnp00429
0.30
GDsnp00336
0.70
GDsnp01541,GDsnp02003
0.70
GDsnp00421,GDsnp00863
0.10
CH01d08
0.60
GDsnp00816,GDsnp01630
0.60
GDsnp00172,GDsnp01562
0.40
GDsnp00273
0.80
GDsnp02029
0.45
MdSNPui01579
1.46
Hi02d02
1.14
MdSNPui00937,MdSNPui02582
0.00
GDsnp01229
1.48
MdSNPui05417
0.33
GDsnp00932
0.90
GDsnp01265
0.66
CTG1064315
0.10
CTG1063606
0.89
GDsnp01412,GDsnp01838
0.30
GDsnp00722
0.70
GDsnp01518
0.20
GDsnp01534
1.00
GDsnp00615
0.50
GDsnp02579
0.90
GDsnp02080
0.04
Hi04c05
0.19
GDsnp00972,GDsnp01622
1.00
GDsnp00281,GDsnp02313
0.30
GDsnp02000,GDsnp02377
0.40
GDsnp01802
0.06
Hi15c07
0.23
GDsnp01599
1.00
GDsnp02823
0.10
GDsnp00458,GDsnp01813
4.80
GDsnp00984
0.29
CTG1068739
0.18
MdSNPui11885
0.13
GDsnp00038
4.66
CN948659
0.56
GDsnp02859
0.30
GDsnp00032
0.40
GDsnp00622,GDsnp00692,GDsnp00848,GDsnp01850
0.50
GDsnp00988
0.70
GDsnp00208
1.30
GDsnp02592,snpEB117266
0.50
GDsnp00962
0.50
GDsnp00983,GDsnp02219
0.81
NB111a
0.37
GDsnp02844
0.10
GDsnp02628
0.10
GDsnp02785
2.50
GDsnp01019,GDsnp02084
0.50
CH02d11
3.29
CH03h06
0.22
GDsnp00241,GDsnp01897,GDsnp01973
0.48
CN908658
2.19
CTG1073847
0.28
GDsnp00454,GDsnp01417
0.00
MdSNPui03614
0.89
Hi21g05y
0.67
GDsnp00772,GDsnp02068,GDsnp02417
0.25
CTG1070919
0.91
GDsnp00582,GDsnp00786,GDsnp01247,GDsnp01347,GDsnp02275,GDsnp02665
0.34
MdSNPui09964,MdSNPui09965,MdSNPui09966
1.12
Hi02f06
1.20
CH04g10,Hi01c06
1.10
CH03b10,GDsnp00357,GDsnp00763
5.70
CTG1064914
1.12
CN913419
0.57
Hi09f01
0.20
CTG1070125
1.38
CTG1070264
1.40
GDsnp01055
0.06
TsuENH016
0.41
GDsnp01133
0.20
GDsnp00129
0.10
MdSNPui02559
0.05
GDsnp01061
2.28
MdSNPui05357
2.81
GDsnp01971
0.80
GDsnp01687,GDsnp01981,GDsnp02231,GDsnp02455
1.20
GDsnp01805
2.00
GDsnp02045
0.70
GDsnp01752
0.43
CN888692,CTG1060546
1.34
CH02c09
0.40
GDsnp00852
0.10
KB08-H5-490/502
0.20
GDsnp01707
1.99
CTG1057509
0.41
GDsnp00661
0.45
CTG1057511
0.25
GDsnp00079
0.11
CN884916-2
0.59
GDsnp00236,GDsnp00436
0.30
GDsnp01389,GDsnp02066
0.50
GDsnp01774,GDsnp01875
1.50
GDsnp00525
2.10
GDsnp01392,GDsnp01963
1.79
KB02-F5
0.21
GDsnp00771,Hi23g12b
0.50
GDsnp02090
0.20
GDsnp01778
0.10
GDsnp02660
1.20
GDsnp01340

LG16

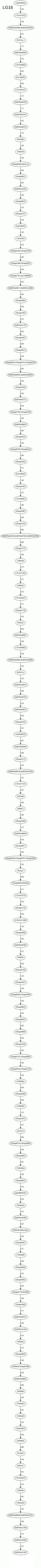

LG17

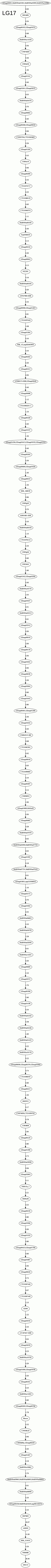
GDsnp00091,MdSNPui01501,MdSNPui03595,MdSNPui10586
0.00
Hi01d06
0.54
GDsnp00192,GDsnp01974
0.68
MdSNPui11018
0.56
CN876284
0.46
CH04c06
1.30
GDsnp01241
0.40
GDsnp01841,GDsnp02075
0.03
MdSNPui04476
0.70
GDsnp00864
0.40
GDsnp00268,GDsnp00818
0.66
CN995784,CTG1063605
0.26
GDsnp01205
0.43
CH04c10
0.66
GDsnp00290
0.32
CTG1074717
0.25
CTG1068125
0.65
CTG1064574
1.11
MdSNPui00165
0.30
TsuENH033
0.13
GDsnp00210
0.20
GDsnp00963
0.13
NZ23f1
0.16
MdSNPui00164
0.20
AF527800-SSR
0.32
GDsnp00508,GDsnp01403
0.81
CTG1057618
1.08
GDsnp01584
0.10
Fdh_1/2,snpEE663955
0.10
GDsnp00202
0.20
GDsnp00313
0.50
AT000174-SSR,GDsnp02049
0.40
GDsnp00088
1.12
CTG1060251-1
1.33
GDsnp00328
0.40
GDsnp01537
1.30
GDsnp01456,GDsnp01613,GDsnp01932,GDsnp02703
0.40
GDsnp00262
0.30
GDsnp00606,GDsnp01626
1.40
GDsnp00515
1.30
SSR_166935
0.86
CH05g03
0.70
AJ001681-SSR
0.10
MdSNPui00418
1.15
CTG1076114
0.35
CH02g04
0.60
CH01h01
1.60
GDsnp01542,GDsnp02550
0.40
MdSNPui10719
1.93
GDsnp01927
0.51
MdSNPui02213
0.61
GDsnp02512
0.50
GDsnp00919
2.00
GDsnp00178
0.50
GDsnp01842
0.90
GDsnp00039
0.30
GDsnp02004
0.60
GDsnp01042
0.80
GDsnp00401,GDsnp01269
0.30
GDsnp01044
0.20
CN894515-289
0.68
CTG1062301
0.92
GDsnp00918
0.05
CTG1059865
0.19
GDsnp00287
0.14
CN858293
1.90
GDsnp01800,Hi03c05
0.50
GDsnp00682
0.28
MdSNPui07970
0.02
MdSNPui02590,MdSNPui07752
0.02
GDsnp01595
0.18
MdSNPui07751,MdSNPui07919
0.01
GDsnp01801,snpGO506943
1.10
GDsnp02172
0.70
GDsnp01904
0.52
MdSNPui06693
0.10
MdSNPui04578
2.28
MdSNPui02950
0.01
MdSNPui11831
0.20
GDsnp00809
2.10
GDsnp02572
1.70
GDsnp00358
2.60
GDsnp01438
0.34
MdSNPui02162
0.38
MdSNPui02436
1.69
MdSNPui01415
0.22
MdSNPui10176
0.22
GDsnp00058,GDsnp00379,GDsnp01964
0.74
CTG1066545
0.60
GDsnp00413
1.20
Hi02f12
0.17
CN876850,CTG1059702
0.78
CH04f08
2.84
GDsnp00125
0.80
GDsnp01298
0.73
MdSNPui03958
0.96
GDsnp02083
0.11
NH013a_1
0.21
Hi05c06
0.22
GDsnp00235
0.80
GDsnp01966
0.50
GDsnp01525
0.80
GDsnp00423,GDsnp01790
0.60
GDsnp01887
0.50
GDsnp00618
0.74
CTG1067449
0.07
CTG1067448
2.15
GD96
1.22
GDsnp02534
0.10
AY187627-SSR
3.04
GDsnp02678
0.23
MdSNPui10755
0.29
GDsnp01098,GDsnp01930
0.20
GDsnp00341
0.43
MdSNPui11052
0.60
GDsnp00265,GDsnp00798
1.78
Slocus
0.32
CN938125
0.96
CH05d08y,GDsnp00245
0.20
GDsnp01467
0.18
MdSNPui10861
2.79
MdSNPui05863,MdSNPui06903,MdSNPui06904
0.01
MdSNPui06900
0.35
GDsnp00438,MdSNPui01610,snpEB140474
0.10
Hi07h02
10.07
145955
11.00
SSR_213431
4.26
NH008b
35.99
246114
